# Supplementary material for: Therapeutic benefit of idebenone in patients with Leber hereditary optic neuropathy: The LEROS nonrandomized controlled trial
Source: Cell Rep Med. 2024 Feb 29;5(3):101437. doi: 10.1016/j.xcrm.2024.101437 (PMC10982982; doi:10.1016/j.xcrm.2024.101437)
Supplement: Document S1. Figures S1–S7 and Tables S1–S10 [file mmc1.pdf]

Cell Reports Medicine, Volume *5*

Supplemental information

Therapeutic benefit of idebenone in patients with Leber hereditary optic neuropathy: The LEROS nonrandomized controlled trial

Patrick Yu-Wai-Man, Valerio Carelli, Nancy J. Newman, Magda Joana Silva, Aki Linden, Gregory Van Stavern, Jacek P. Szaflik, Rudrani Banik, Wojciech Lubiński, Berthold Pemp, Yaping Joyce Liao, Prem S. Subramanian, Marta Misiuk-Hojło, Steven Newman, Lorena Castillo, Jarosław Kocięcki, Marc H. Levin, Francisco Jose Muñoz-Negrete, Ali Yagan, Sylvia Cherninkova, David Katz, Audrey Meunier, Marcela Votruba, Magdalena Korwin, Jacek Dziedziak, Neringa Jurkutė, Joshua P. Harvey, Chiara La Morgia, Claudia Priglinger, Xavier Llòria, Livia Tomasso, Thomas Klopstock, and LEROS Study Group

Supplemental Information

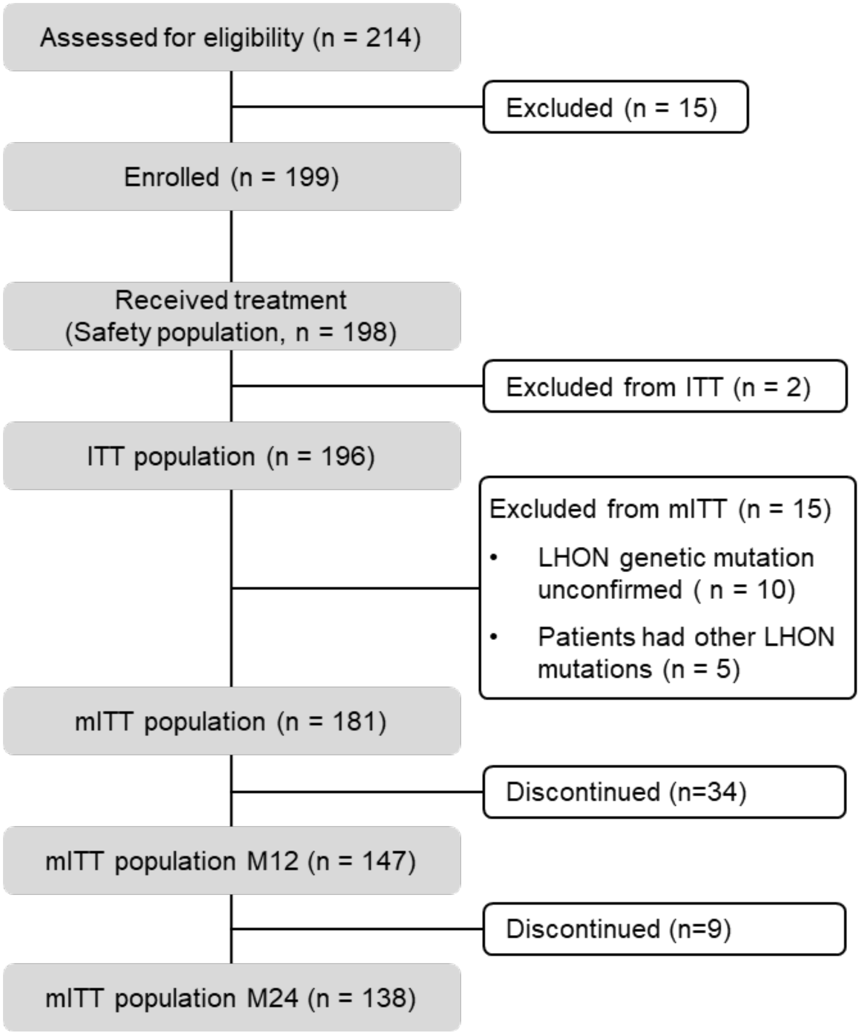

Figure S 1. Disposition of idebenone treated patients, related to Results and STAR Methods

ITT: Intention-To-Treat; LHON: Leber hereditary optic neuropathy; mITT: modified ITT; VA: visual acuity.

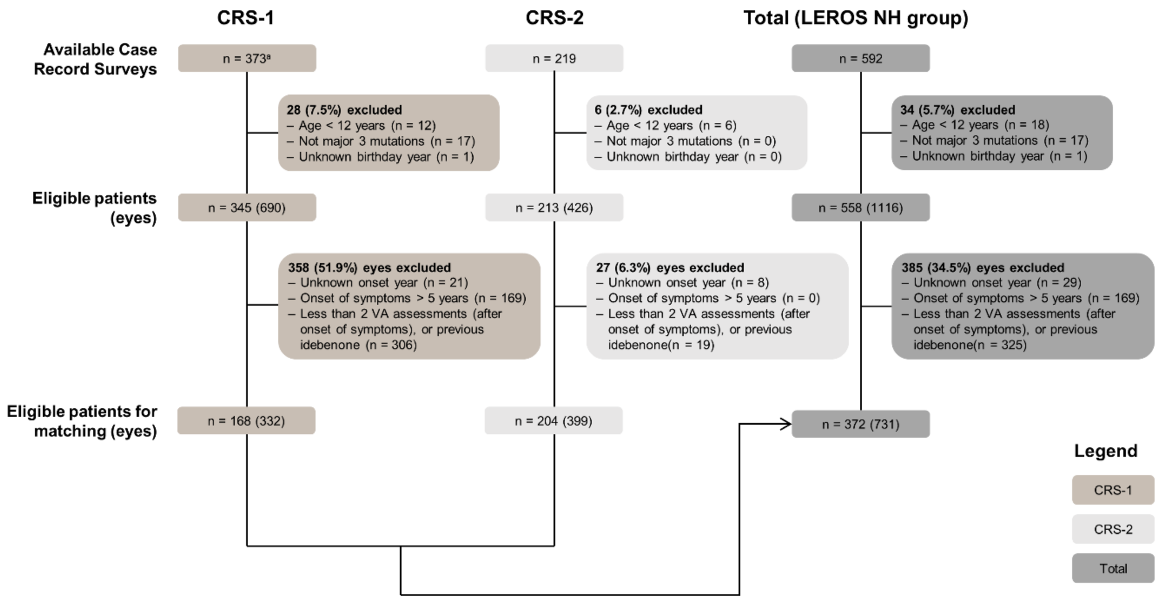

Figure S 2. Disposition of patients in the Natural History control group, related to STAR Methods
[a]Of 383 available record surveys, 10 were of patients included in CRS-2 and were removed from CRS-1. CRS: Case Record Survey; NH: Natural History.

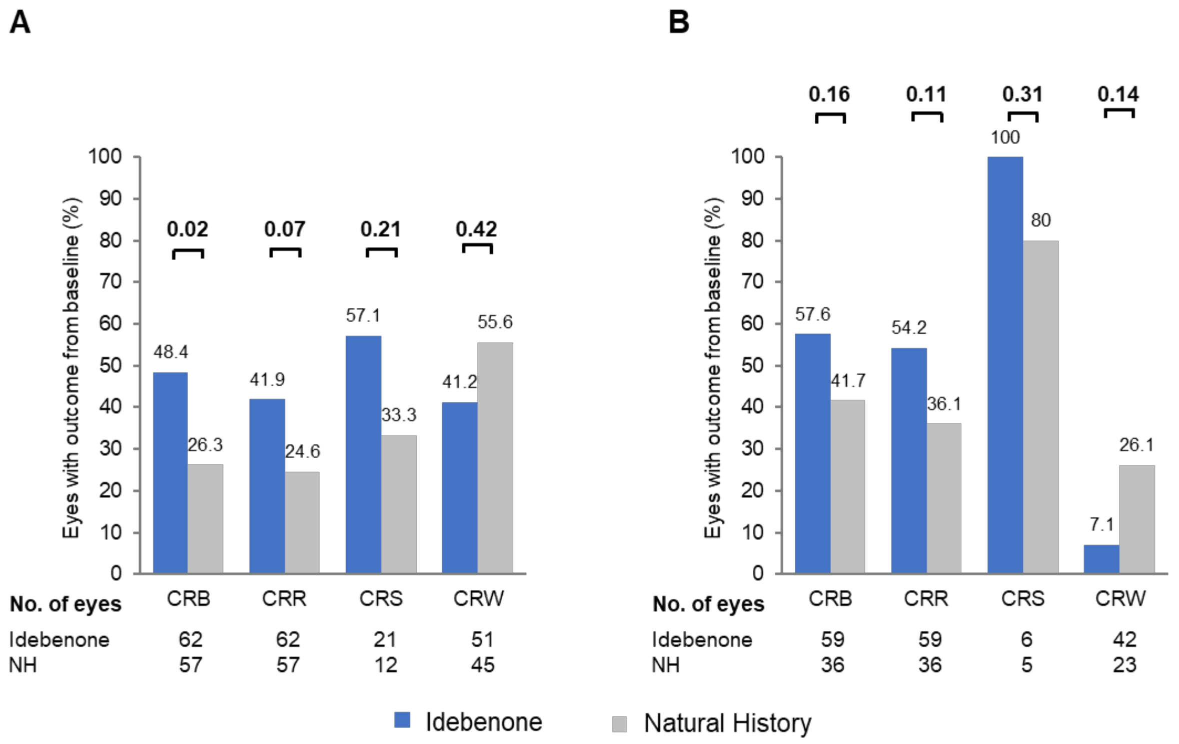

Figure S 3. Responder outcomes at 24 months in (A) subacute eyes (< 6 months after onset at baseline) and (B) dynamic eyes (6 – 12 months after onset at baseline) (mITT vs matched NH). Related to Figure 1
CRB: clinically relevant benefit; CRS: clinically relevant stabilization; CRR: clinically relevant recovery; CRW: clinically relevant worsening; mITT: modified Intention-To-Treat cohort; NH: Natural History cohort.

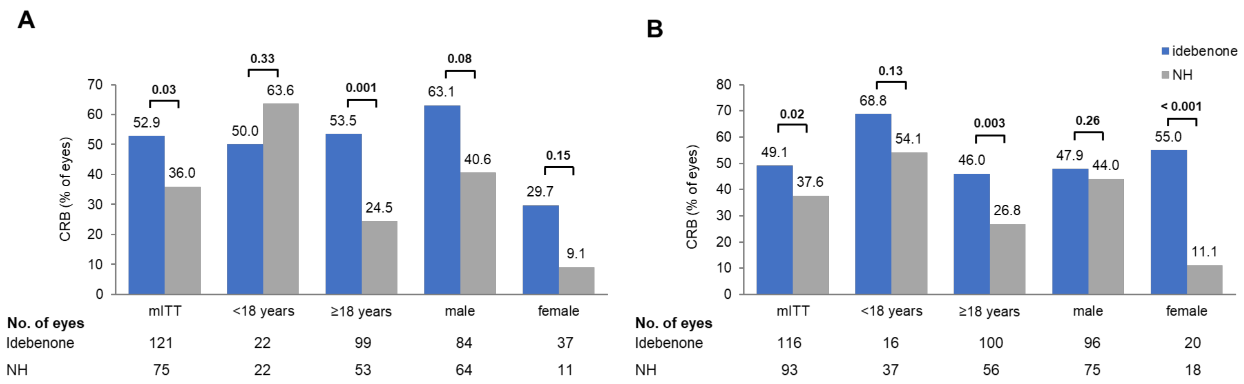

Figure S 4. CRB from baseline by age group at first symptom onset and by gender in (A) subacute/dynamic and (B) chronic eyes at 24 months (mITT vs matched NH). Related to Figure 1
Idebenone had a significant therapeutic benefit in adult eyes (≥18 years) at 24 months (subacute/dynamic [53.5% vs 24.5%, p=0.001]; chronic [46.0% vs 26.8%, p=0.003]). In patients <18 years at symptom onset, the rate of CRB was comparably high in both treated and untreated eyes (subacute/dynamic [50.0% vs 63.6%, p=0.33]; chronic [68.8% vs 54.1%, p=0.13]). Subgroup analyses of CRB by ***gender*** demonstrated a therapeutic benefit of idebenone at 24 months in eyes of female patients in the chronic phase. In the subacute/dynamic phase there was a non-significant trend toward a positive treatment effect in both ***genders***. In subacute/dynamic eyes, the difference in rates was larger in eyes of male patients, whereas this was reversed in the chronic phase. CRB: clinically relevant benefit; mITT: modified Intention-To-Treat cohort; NH: Natural History cohort.

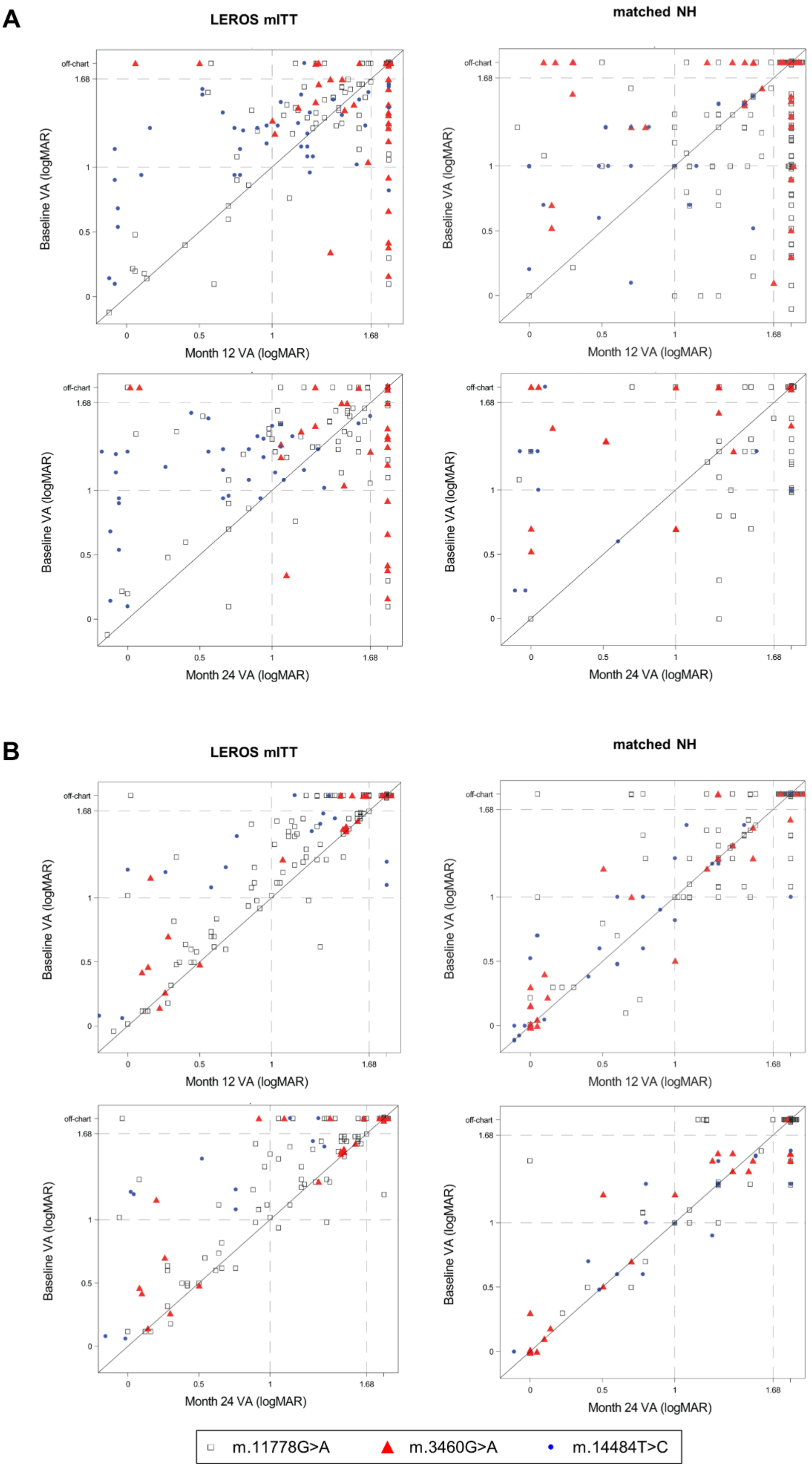

Figure S 5. Visual acuity of individual eyes at baseline versus 12 or 24 months in (A) subacute/dynamic eyes, and (B) chronic eyes (mITT and matched Natural History groups). Related to Figure 4
Off-chart VA was defined as 1.8 logMAR. mITT: modified intention-to-treat; NH: Natural History; VA: visual acuity.

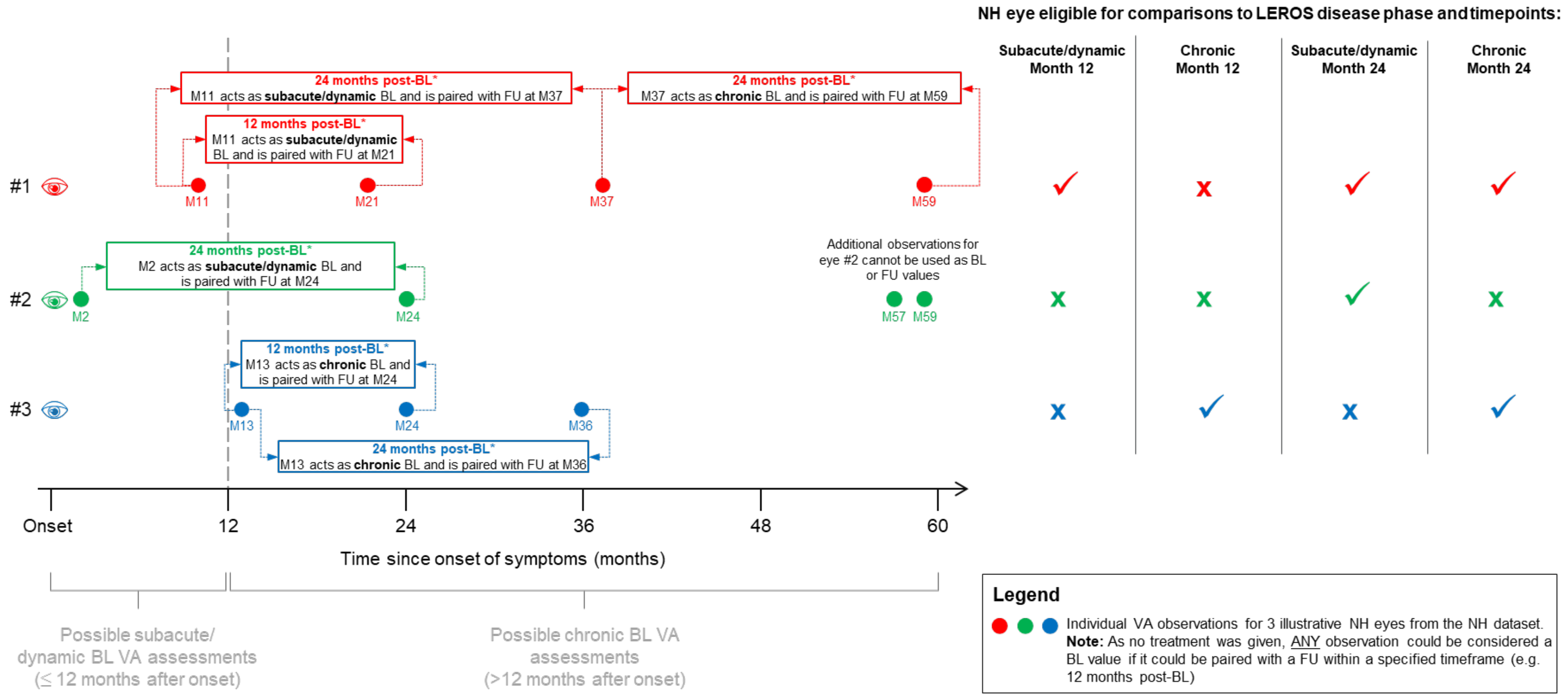

Figure S 6. Illustration of matching subacute/dynamic eyes in the Natural History group to the idebenone-treated LEROS mITT population, related to STAR Methods

* 12 or 24 months post-BL ± 3 months.

BL: baseline; FU: follow-up; NH: Natural History; M: Month; VA: visual acuity.

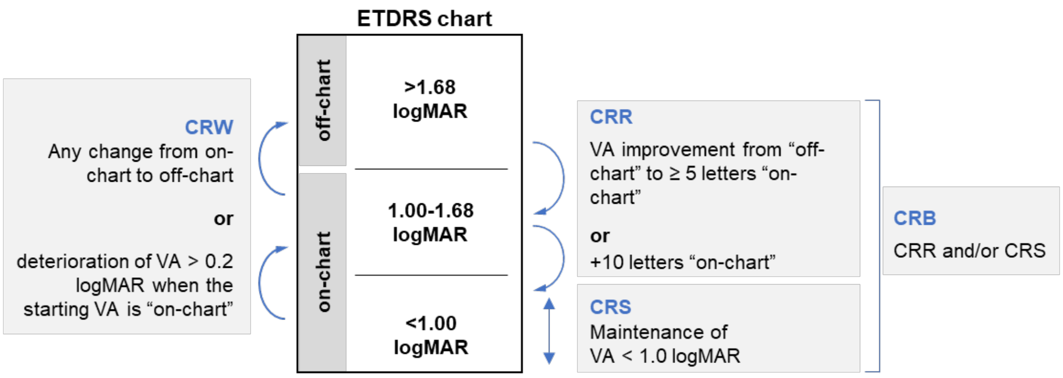

Figure S 7. Definition of outcome measures, related to STAR Methods
CRB: clinically relevant benefit; CRR: clinically relevant recovery; CRS: clinically relevant stabilization; CRW: clinically relevant worsening; ETDRS: Early Treatment Diabetic Retinopathy Study; VA: visual acuity.

Table S 1. Patient characteristics in the different Natural History subsets, related to Table 1

In line with literature reports, around 80% of patients were male with a median age of first symptom onset around 24 – 26 years. [1] The proportions of the three major mutations were also as expected with m.11778G>A found in 73% of patients, followed by 14% carrying the m.3460G>A mutation, and 13% the m.14484T>C mutation in patients eligible for matching.(Yu-Wai-Man et al. 2009) At first symptom onset, 4.6% of patients eligible for matching were <12 years of age and 15.6% were adolescent (12 to <18 years). The majority of patients were adults, with 46.5% in the age group of 18 to <35 years and 33.3% in the age group of 35 years or older. For over 50% of patients eligible for matching at least 5 visits with VA assessments were recorded. Nevertheless, the number of visits was relatively low, leading to a strong reduction of patients with possible baseline-visit pairs for the outcome analyses. Only 36.0% (n=134) of patients eligible for matching had a suitable VA pair at 12 months, and 18.5% (n=69) at 24 months.

Characteristic	(A) Total NH population	(B) Patients with ≥ 2 visits prior to idebenone	(C) Patients in (B) with known onset year and ≤ 5 years since onset	(D) Patients in (C) with one of major 3 mutations	(E) Patients eligible for matching (Patients in (D) aged ≥ 12 years and year of birth known)
**Patients, N**	**592**	**419**	**391**	**383**	**372**
**Gender, n (%)**					
Female	124 (20.9%)	85 (20.3%)	78 (19.9%)	76 (19.8%)	**74 (19.9%)**
Male	462 (78.0%)	333 (79.5%)	312 (79.8%)	306 (79.9%)	**297 (79.8%)**
Missing	6 (1.0%)	1 (0.2%)	1 (0.3%)	1 (0.3%)	**1 (0.3%)**
**Mutation, n (%)**					
m.11778G>A	404 (68.2%)	295 (70.4%)	279 (71.4%)	279 (72.8%)	**273 (73.4%)**
m.3460G>A	95 (16.0%)	59 (14.1%)	55 (14.1%)	55 (14.4%)	**52 (14.0%)**
m.14484T>C	76 (12.8%)	54 (12.9%)	49 (12.5%)	49 (12.8%)	**47 (12.6%)**
Other	17 (2.9%)	11 (2.6%)	8 (2.0%)	-	**-**
**Age (years) at 1st symptom onset (years), N**	581	413	390	382	**372**
Mean (SD)	28 (14.6)	29 (14.6)	29 (14.6)	29 (14.6)	**30 (14.3)**
Median (Q1; Q3)	24 (17; 37)	25 (18; 38)	25 (18; 39)	26 (18; 39)	**26 (18; 39)**
Min – max	4, 78	4, 75	4, 75	4, 75	**4, 75**
**Age group at 1st symptom onset, n (%)**					
<12 years	44 (7.4%)	33 (7.9%)	28 (7.2%)	27 (7.0%)	**17 (4.6%)**
12 ≤ years <18	102 (17.2%)	69 (16.5%)	60 (15.3%)	58 (15.1%)	**58 (15.6%)**
18 ≤ years <35	270 (45.6%)	183 (43.7%)	177 (45.3%)	173 (45.2%)	**173 (46.5%)**
≥ 35 years	165 (27.9%)	128 (30.5%)	125 (32.0%)	124 (32.4%)	**124 (33.3%)**
Missing	11 (1.9%)	6 (1.4%)	1 (0.3%)	1 (0.3%)	**-**
**Number of visits per patient with VA assessment, N**	587	419	391	383	**372**
Mean (SD)	4 (3.5)	5 (3.6)	6 (3.6)	5 (3.6)	**5 (3.6)**
Median (Q1; Q3)	3 (2; 6)	4 (3; 7)	5 (3; 7)	5 (3; 7)	**5 (3; 7)**
Range	1, 31	2, 31	2, 31	2, 31	**2, 31**
1	109 (18.4%)	-	-	-	**-**
2	104 (17.6%)	79 (18.9%)	70 (17.9%)	70 (18.3%)	**68 (18.3%)**
3	94 (15.9%)	81 (19.3%)	74 (18.9%)	74 (19.3%)	**70 (18.8%)**
4	56 (9.5%)	50 (11.9%)	47 (12.0%)	46 (12.0%)	**45 (12.1%)**
5	58 (9.8%)	55 (13.1%)	53 (13.6%)	52 (13.6%)	**50 (13.4%)**
>5	166 (28.0%)	154 (36.8%)	147 (37.6%)	141 (36.8%)	**139 (37.4%)**

SD: standard deviation; Q: quartile; VA: visual acuity.

Table S 2. Follow-up time and time in NH patient population, related to Table 1

	Patients eligible for matching (N=372)
**Follow-up time (months)**	
Mean (SD)	47.9 (72.41)
Median (Q1; Q3)	14.7 (4.6; 67.6)
Range	0.1 – 514.1
**Time since onset of symptoms to first visit (years)**	
Mean (SD)	0.4 (0.91)
Median (Q1; Q2)	0.1 (0.0; 0.4)
Range	0.0 – 11.2
≤1 year since onset, n (%)	337 (90.6)
>1 year since onset, n (%)	35 (9.4)

Q: quartile; SD: standard deviation; VA: visual acuity.

Table S 3. Visual acuity reporting formats in the Natural History dataset eligible for matching, related to STAR Methods

	**Patients eligible for matching (N=372)**
Type of VA recording, F	3999
logMAR, f (%)	509 (12.7)
Off-chart[a], f (%)	804 (20.1)
Decimal, f (%)	579 (14.5)
Snellen, f (%)	2052 (51.3)
Other, f (%)	55 (1.4)

[a] Counting fingers or worse. Off-chart VA was converted to 1.8 logMAR.
F: frequency; f: frequency; VA: visual acuity.

Table S 4. Patient baseline demographics and characteristics in subacute/dynamic and chronic patients (ITT), related to Table 1

Characteristic	Subacute/dynamic patients (N=109)	Chronic patients (N=87)	ITT (N=196)
Mutation, n (%)	109	87	196
m.11778G>A	55 (50.5)	57 (65.5)	112 (57.1)
m.3460G>A	20 (18.3)	15 (17.2)	35 (17.9)
m.14484T>C	24 (22.0)	10 (11.5)	34 (17.3)
Other	2 (1.8)	3 (3.4)	5 (2.6)
No identified mutation	8 (7.3)	2 (2.3)	10 (5.1)
Male, n (%)	78 (71.6)	66 (75.9)	144 (73.5)
Childbearing potential, N	31	21	52
Yes, n (%)	22 (71.0)	13 (61.9)	35 (67.3)
Race, N	109	87	196
Black or African American, n (%)	6 (5.5)	2 (2.3)	8 (4.1)
White, n (%)	36 (33.0)	18 (20.7)	54 (27.6)
Other[a], n (%)	67 (61.5)	67 (76.9)	134 (68.3)
Height (cm), N	104	85	189
Mean (SD)	172.19 (10.47)	170.44 (12.11)	171.41 (11.24)
Min – max	123.50 – 195.01	107.18 – 190.50	107.18 – 195.01
Weight (kg), N	104	85	189
Mean (SD)	74.54 (15.72)	72.74 (15.79)	73.73 (15.73)
Min – max	43.40 – 119.75	34.10 – 115.21	34.10 – 119.75
Age (years), N	109	87	196
Mean (SD)	34.05 (15.06)	34.06 (15.49)	34.06 (15.21)
Min – max	12.56 – 79.23	12.13 – 76.88	12.13 – 79.23
Age at 1st symptom onset (years), N	109	87	196
Mean (SD), years	33.42 (14.98)	31.43 (15.58)	32.53 (15.24)
Min – max	12.05 – 78.17	8.75 – 75.75	8.75 – 78.17
Months since most recent symptom onset, N	109	87	196
Mean (SD)	5.32 (3.04)	30.30 (14.22)	16.41 (15.78)
Min – max	0.07 – 11.86	12.06 – 57.95	0.07 – 57.95
Number of symptomatic eyes per patient, N	109	87	196
One, n (%)	8 (7.3)	3 (3.4)	11 (5.6)
Two, n (%)	101 (92.7)	84 (96.6)	185 (94.4)
Simultaneous onset in both eyes, N	109	87	196
n (%)	41 (37.6)	37 (42.5)	78 (39.8)
Delta onset[b] (months), N	65	47	112
Mean (SD)	4.49 (5.43)	2.57 (2.42)	3.69 (4.51)
Min – max	0.03 – 27.96	0.30 – 11.86	0.03 – 27.96

[a]Other races included 1 (0.9%) Chinese in the subacute/dynamic LHON cohort, and 2 (2.3%) American Indian or Alaska Native, 1 (1.1%) Asian Indian, and 1 (1.1%) Filipino in the chronic LHON cohort. For 66 (60.6%) patients in the subacute/dynamic and 63 (72.4%) in the chronic LHON cohort the race was not specified.[b]Delta onset is the time gap between symptom onset between a patient's two eyes. Eyes that have equal dates onset were not accounted in the calculation of the delta. ITT: intention-to-treat; LHON: Leber hereditary optic neuropathy; max: maximum; min: minimum; SD: standard deviation.

Table S 5. Responder analysis outcomes in subacute/dynamic and chronic eyes (mITT vs matched NH group), related to Figure 1
The sensitivity analysis takes into consideration additional covariates of age at first symptom onset and time since symptom onset, in addition to treatment, mutations, and gender.

	**All (mITT)**							
	**12 months**				**24 months**			
	**ide,** n/N (%)	**NH,** n/N (%)	***p*-Value; OR [95% CI]**	***Sensitivity analysis* *p*-Value; OR [95% CI]**	**ide,** n/N (%)	**NH,** n/N (%)	***p*-Value; OR [95% CI]**	***Sensitivity analysis* *p*-Value; OR [95% CI]**
**SUBACUTE/DYNAMIC EYES**								
CRB	60/142 (42.3)	40/193 (20.7)	0.002; 2.29 [1.35; 3.88]	0.008; 2.10 [1.22; 3.64]	64/121 (52.9)	27/75 (36.0)	0.030; 2.08 [1.07; 4.10]	0.048; 1.96 [1.01; 3.89]
CRR	47/142 (33.1)	35/193 (18.1)	0.087; 1.65 [0.93; 2.92]	0.215; 1.45 [0.80; 2.62]	58/121 (47.9)	25/75 (33.3)	0.068; 1.86 [0.96; 3.66]	0.113; 1.73 [0.88; 3.44]
CRS	20/31 (64.5)	9/40 (22.5)	<0.001; 7.32 [2.34; 25.91]	0.001; 7.80 [2.17; 33.84]	18/27 (66.7)	6/13 (46.2)	0.096; 5.23 [0.76; 52.91]	0.037; 12.53 [1.15; 375.2]
CRW	30/103 (29.1)	76/130 (58.5)	<0.001; 0.33 [0.18; 0.61]	0.023; 0.45 [0.22; 0.89]	24/93 (25.8)	25/49 (51.0)	0.005; 0.30 [0.12; 0.70]	0.009; 0.29 [0.11; 0.74]
**CHRONIC EYES**								
CRB	72/143 (50.3)	59/153 (38.6)	0.009; 1.93 [1.18; 3.17]	0.009; 1.94 [1.18; 3.21]	57/116 (49.1)	35/93 (37.6)	0.018; 2.05 [1.13; 3.79]	0.022; 2.05 [1.11; 3.87]
CRR	47/143 (32.9)	30/153 (19.6)	0.003; 2.24 [1.30; 3.93]	0.006; 2.16 [1.25; 3.80]	37/116 (31.9)	15/93 (16.1)	0.001; 3.15 [1.55; 6.77]	0.005; 2.84 [1.36; 6.24]
CRS	32/34 (94.1)	36/38 (94.7)	0.894; 0.85 [0.06; 10.23]	0.919; 0.86 [0.04; 15.18]	26/28 (92.9)	22/23 (95.7)	0.870; 0.75 [0.01; 20.38]	0.908; 0.55 [0.00; 3372]
CRW	4/81 (4.9)	15/89 (16.9)	0.006; 0.22 [0.06; 0.66]	0.016; 0.26 [0.07; 0.78]	2/68 (2.9)	12/60 (20.0)	<0.001; 0.08 [0.01; 0.34]	<0.001; 0.09 [0.01; 0.40]

CI: confidence interval.; CRB: clinically relevant benefit; CRR: clinically relevant recovery; CRS: clinically relevant stabilization; CRW: clinically relevant worsening; ide: idebenone; mITT: modified Intention-To-Treat; NH: Natural History cohort; OR: odds ratio.

Table S 6: Logistic regression Type III tests of Fixed Effects for CRB at 12 and 24 months in subacute/dynamic and chronic eyes by mutation (mITT vs matched NH group), related to Figure 1

**Interaction model**	**Month 12 *p*-values**	**Month 24 *p*-values**
**Subacute/dynamic eyes**		
Interaction treatment and major 3 mutations	0.0015	0.0078
Treatment	0.0788	0.4529
Mutations	<.0001	<.0001
Gender	0.1726	0.0021
**Chronic eyes**		
Interaction treatment and major 3 mutations	0.0242	0.0260
Treatment	0.5716	0.9798
Mutations	<.0001	0.0232
Gender	0.3358	0.3243

Table S 7. Responder analysis outcomes in subacute/dynamic and chronic eyes by mutation (mITT vs matched NH group), related to Figure 1

	m.11778G>A						m.3460G>A						m.14484T>C					
	12 months			24 months			12 months			24 months			12 months			24 months		
	**Ide,** n/N (%)	**NH,** n/N (%)	***p*-Value; Odds Ratio [95% CI]**	**Ide** n/N (%)	**NH,** n/N (%)	***p*-Value; Odds Ratio [95% CI]**	**Ide,** n/N (%)	**NH,** n/N (%)	***p*-Value; Odds Ratio [95% CI]**	**Ide,** n/N (%)	**NH,** n/N (%)	***p*-Value; Odds Ratio [95% CI]**	**Ide,** n/N (%)	**NH,** n/N (%)	***p*-Value; Odds Ratio [95% CI]**	**Ide,** n/N (%)	**NH,** n/N (%)	***p*-Value; Odds Ratio [95% CI]**
**SUBACUTE/DYNAMIC EYES**																		
CRB	25/68 (36.8)	13/138 (9.4)	<0.001; 6.01 [2.85; 13.24]	27/60 (45.0)	8/47 (17.0)	<0.001; 5.30 [2.12; 14.49]	12/32 (37.5)	14/34 (41.2)	1.0; 1.00 [0.36; 2.77]	9/26 (34.6)	11/18 (61.1)	0.24; 0.47 [0.12; 1.67]	23/42 (54.8)	13/21 (61.9)	0.60; 0.75 [0.25; 2.18]	28/35 (80.0)	8/10 (80.0)	0.98; 1.02 [0.13; 5.52]
CRR	14/68 (20.6)	10/138 (7.2)	0.005; 3.54 [1.48; 8.75]	23/60 (38.3)	7/47 (14.9)	0.002; 4.51 [1.75; 12.84]	12/32 (37.5)	14/34 (41.2)	0.99; 1.00 [0.36; 2.77]	9/26 (34.6)	11/18 (61.1)	0.22; 0.45 [0.12; 1.61]	21/42 (50.0)	11/21 (52.4)	0.87; 0.92 [0.32; 2.64]	26/35 (74.3)	7/10 (70.0)	0.77; 1.27 [0.23; 5.87]
CRS	12/15 (80.0)	3/28 (10.7)	<0.001; 37.03 [7.13; 283.2]	9/12 (75.0)	1/6 (16.7)	<0.001; 127E4 [6.84; N.E.]	0/6 (0.0)	2/6 (33.3)	0.10; 0.00 [N.E.; 1.81]	0/6 (0.0)	2/4 (50.0)	0.09;0.00 [N.E.; 1.70]	8/10 (80.0)	4/6 (66.7)	0.63; 1.78 [0.15; 20.80]	9/9 (100.0)	3/3 (100.0)	N.E.; 1.0 [N.E.; N.E.]
CRW	10/45 (22.2)	64/92 (69.6)	<0.001; 0.13 [0.05; 0.28]	10/41 (24.4)	20/30 (66.7)	<0.001; 0.11 [0.03; 0.34]	14/20 (70.0)	9/17 (52.9)	0.28; 2.10 [0.54; 8.59]	13/17 (76.5)	3/10 (30.0)	0.05; 5.63 [1.02; 38.94]	6/38 (15.8)	3/21 (14.3)	0.88; 1.12 [0.26; 5.83]	1/35 (2.9)	2/9 (22.2)	0.07; 0.10 [0.00; 1.19]
**CHRONIC EYES**																		
CRB	50/105 (47.6)	24/102 (23.5)	<0.001; 2.93 [1.63; 5.40]	36/82 (43.9)	11/51 (21.6)	0.008; 2.85 [1.31; 6.56]	10/23 (43.5)	16/26 (61.5)	0.24; 0.51 [0.16; 1.58]	10/23 (43.5)	16/24 (66.7)	0.12; 0.40 [0.12; 1.28]	12/15 (80.0)	19/25 (76.0)	0.81; 1.21 [0.26; 6.60]	11/11 (100.0)	8/18 (44.4)	<0.001; 462E4 [4.86; N.E.]
CRR	29/105 (27.6)	17/102 (16.7)	0.06; 1.90 [0.97; 3.78]	20/82 (24.4)	7/51 (13.7)	0.13; 2.03 [0.82; 5.54]	7/23 (30.4)	6/26 (23.1)	0.52; 1.52 [0.42; 5.64]	7/23 (30.4)	5/24 (20.8)	0.43; 1.70 [0.45; 6.78]	11/15 (73.3)	7/25 (28.0)	0.006; 6.86 [1.73; 32.34]	10/11 (90.9)	3/18 (16.7)	<0.001; 46.98 [5.90; 1065]
CRS	24/26 (92.3)	9/9 (100.0)	0.23; 0.00 [N.E.; 4.00]	18/20 (90.0)	4/4 (100.0)	0.45; 0.00 [N.E.; 13.59]	6/6 (100.0)	12/13 (92.3)	0.29; 30790 [0.11; N.E.]	6/6 (100.0)	12/12 (100.0)	N.E.; 1.29 [N.E.; N.E.]	2/2 (100.0)	15/16 (93.8)	0.7156; 14525 [0.01; N.E.]	2/2 (100.0)	6/7 (85.7)	0.54; 44571 [0.02; N.E.]
CRW	2/55 (3.6)	11/44 (25.0)	0.002; 0.11 [0.02; 0.46]	2/45 (4.4)	6/19 (31.6)	0.004; 0.10 [0.01; 0.49]	0/15 (0.0)	3/21 (14.3)	0.05; 0.00 [N.E.; 1.00]	0/15 (0.0)	3/23 (13.0)	0.05; 0.00 [N.E.; 1.03]	2/11 (18.2)	1/24 (4.2)	0.17; 5.55 [0.47; 129.9]	0/8 (0.0)	3/18 (16.7)	0.21; 0.00 [N.E.; 3.29]

CRB: clinically relevant benefit; CRR: clinically relevant recovery; CRS: clinically relevant stabilization; CRW: clinically relevant worsening; ide: idebenone; mITT: modified Intention-To-Treat; N.E.: not estimable; NH: Natural History cohort.

Table S 8. Visual acuity (logMAR) at baseline and in the 12- and 24-month cohorts (by eyes; mITT vs matched NH group), related to Figure 4

	**Total**		**m.11778G>A**		**m.3460G>A**		**m.14484T>C**	
	**Idebenone**	**NH**	**Idebenone**	**NH**	**Idebenone**	**NH**	**Idebenone**	**NH**
**Subacute/dynamic eyes, 12 months**								
N	142	193	68	138	32	34	42	21
**VA at baseline, logMAR**								
LS-Means (SE)	1.29 (0.04)	1.26 (0.05)	1.29 (0.06)	1.27 (0.05)	1.35 (0.09)	1.46 (0.09)	1.23 (0.08)	1.02 (0.11)
95% CI	1.20; 1.38	1.17; 1.36	1.17; 1.42	1.18; 1.37	1.18; 1.53	1.28; 1.64	1.07; 1.38	0.80; 1.23
**VA at 12 months**								
LS-Means (SE)	1.20 (0.04)	1.32 (0.05)	1.25 (0.06)	1.57 (0.04)	1.49 (0.08)	1.27 (0.08)	0.99 (0.08)	0.84 (0.11)
95% CI	1.12; 1.29	1.23; 1.42	1.13; 1.36	1.48; 1.66	1.32; 1.65	1.10; 1.43	0.84; 1.14	0.64; 1.05
**Difference, logMAR (*p*-value)**[a]	-0.12 (0.03)		-0.33 (<0.001)		0.22 (0.06)		0.14 (0.25)	
**Subacute/dynamic eyes, 24 months**								
N	121	75	60	47	26	18	35	10
**VA at baseline, logMAR**								
LS-Means (SE)	1.26 (0.05)	1.30 (0.07)	1.31 (0.06)	1.36 (0.07)	1.29 (0.09)	1.45 (0.12)	1.18 (0.09)	1.03 (0.15)
95% CI	1.17; 1.36	1.17; 1.43	1.19; 1.43	1.21; 1.50	1.11; 1.48	1.22; 1.68	1.01; 1.35	0.73; 1.33
**VA at 24 months, logMAR**								
LS-Means (SE)	1.11 (0.05)	1.14 (0.08)	1.22 (0.07)	1.53 (0.08)	1.48 (0.10)	0.95 (0.13)	0.77 (0.09)	0.57 (0.17)
95% CI	1.00; 1.22	0.98; 1.29	1.08; 1.35	1.37; 1.69	1.28; 1.68	0.70; 1.20	0.58; 0.95	0.23; 0.90
**Difference, logMAR (*p*-value)**[a]	-0.03 (0.75)		-0.32 (0.002)		0.53 (0.001)		0.20 (0.29)	
**Chronic eyes, 12 months**								
N	143	153	105	102	23	26	15	25
**VA at baseline, logMAR**								
LS-Means (SE)	1.16 (0.06)	1.11 (0.06)	1.35 (0.06)	1.49 (0.06)	1.31 (0.11)	0.82 (0.11)	1.25 (0.14)	0.71 (0.11)
95% CI	1.04; 1.27	1.00; 1.22	1.23; 1.46	1.38; 1.60	1.09; 1.53	0.61; 1.04	0.97; 1.53	0.49; 0.92
**VA at 12 months, logMAR**								
LS-Means (SE)	1.11 (0.03)	1.21 (0.03)	1.17 (0.03)	1.27 (0.03)	1.16 (0.06)	1.20 (0.06)	0.97 (0.08)	1.17 (0.06)
95% CI	1.05; 1.18	1.15; 1.28	1.10; 1.23	1.21; 1.34	1.04; 1.29	1.07; 1.33	0.82; 1.13	1.04; 1.30
**Difference, logMAR (*p*-value)**[a]	-0.10 (0.004)		-0.10 (0.02)		-0.04 (0.68)		-0.20 (0.05)	
**Chronic eyes, 24 months**								
N	116	93	82	51	23	24	11	18
**VA at baseline, logMAR**								
LS-Means (SE)	1.20 (0.07)	1.18 (0.07)	1.34 (0.07)	1.54 (0.08)	1.32 (0.11)	0.82 (0.11)	1.21 (0.17)	1.00 (0.13)
95% CI	1.07; 1.33	1.04; 1.31	1.21; 1.47	1.38; 1.69	1.10; 1.54	0.60; 1.04	0.88; 1.54	0.75; 1.25
**VA at 24 months, logMAR**								
LS-Means (SE)	1.07 (0.04)	1.24 (0.04)	1.15 (0.04)	1.26 (0.05)	1.11 (0.06)	1.24 (0.07)	0.75 (0.09)	1.27 (0.07)
95% CI	0.99; 1.15	1.16; 1.31	1.08; 1.23	1.17; 1.36	0.98; 1.24	1.11; 1.37	0.57; 0.94	1.13; 1.42
**Difference, logMAR (*p*-value)**[a]	-0.17 (<0.001)		-0.11 (0.04)		-0.13 (0.16)		-0.52 (<0.001)	

[a]Difference in LS-means VA in change from baseline between idebenone-treated eyes and matched eyes in the NH group. A negative difference indicates a relative improvement in visual acuity in treated eyes compared to the Natural History group.

CI: confidence interval; LS: least squares; mITT: modified Intention-To-Treat cohort; NH: Natural History cohort; SE: standard error; VA: visual acuity.

Table S 9. Safety population baseline demographics, related to Table 1

	**Safety population (N=198)**
Gender, N	198
Male, n (%)	146 (73.7)
Female, n (%)	52 (26.3)
Childbearing potential, N	52
Yes, n (%)	35 (67.3)
No, n (%)	17 (32.7)
Mutation, N	198
m.11778G>A, n (%)	112 (56.6)
m.3460G>A, n (%)	35 (17.7)
m.14484T>C, n (%)	34 (17.2)
Other, n (%)	5 (2.5)
Negative, n (%)	12 (6.1)
Race, N	198
Black or African American, n (%)	9 (4.5)
White, n (%)	54 (27.3)
Other[a], n (%)	135 (68.2)
Height at baseline (cm), N	191
Mean (SD)	171.3 (11.3)
Min – max	107.2 – 195.0
Weight at baseline (kg), N	191
Mean (SD)	73.6 (15.7)
Min – max	34.1 – 119.8
Age at baseline (years), N	198
Mean (SD)	34.2 (15.2)
Min – max	12.13 – 79.2

[a]Other races included 2 (1.0%) American Indian or Alaska Native, 1 (0.5%) Asian Indian, 1 (0.5%) Chinese, and 1 (0.5%) Filipino. For 130 (65.7%) patients the race was not specified.
max: maximum; min: minimum; SD: standard deviation.

Table S 10. Summary of treatment-emergent adverse events (safety population), related to Table 2

	Events	Patients	Days in Treatment	
	f (%) (F=891)	n (%) (N=198)	Mean (SD)	Min – Max
Any TEAEs	891 (100.0)	154 (77.8)	247.0 (234.9)	1.0 – 1027.0
Product-related TEAEs	101 (11.3)	49 (24.7)	120.4 (178.5)	1.0 – 757.0
Severe TEAEs	25 (2.8)	13 (6.6)	325.0 (252.5)	19.0 – 742.0
Serious TEAEs not leading to death	44 (4.9)	27 (13.6)	291.4 (239.7)	14.0 – 742.0
TEAEs (special interest)	79 (8.9)	36 (18.2)	353.5 (291.3)	1.0 – 1027.0
TEAEs leading to permanent study treatment discontinuation	18 (2.0)	10 (5.1)	241.3 (264.6)	14.0 – 742.0
TEAEs leading to death	1 (0.1)	1 (0.5)	517.0 (-)	517.0 – 517.0

max: maximum; min: minimum; SD: standard deviation; TEAE: treatment-emergent adverse event.

REFERENCES

1. Yu-Wai-Man, P.; Griffiths, P. G.; Hudson, G.; Chinnery, P. F. (2009): Inherited mitochondrial optic neuropathies. In *J Med Genet* 46 (3), pp. 145–158. DOI: 10.1136/jmg.2007.054270.
