# Supplementary material for: Therapeutic benefit of idebenone in patients with Leber hereditary optic neuropathy: The LEROS nonrandomized controlled trial
Source: Cell Rep Med. 2024 Feb 29;5(3):101437. doi: 10.1016/j.xcrm.2024.101437 (PMC10982982; doi:10.1016/j.xcrm.2024.101437)
Supplement: Document S2. Article plus supplemental information [file mmc2.pdf]

Cell Reports Medicine

Article

Therapeutic benefit of idebenone in patients with Leber hereditary optic neuropathy: The LEROS nonrandomized controlled trial

Graphical abstract

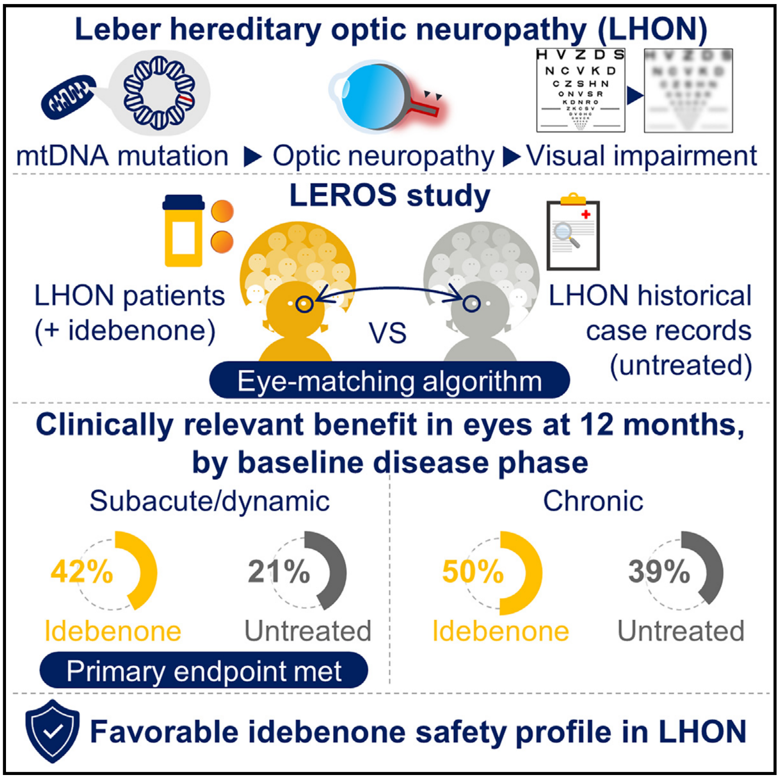

Highlights

- LEROS meets its primary endpoint based on clinically relevant benefit at month 12
- Favorable outcomes are observed to month 24, regardless of disease phase
- Treatment effect is influenced by disease phase, mtDNA mutation, age, and gender
- LEROS confirms a favorable safety profile of idebenone in LHON patients

Authors

Patrick Yu-Wai-Man, Valerio Carelli, Nancy J. Newman, ..., Livia Tomasso, Thomas Klopstock, LEROS Study Group

Correspondence

thomas.klopstock@med.uni-muenchen.de

In brief

Yu-Wai-Man et al. demonstrate how idebenone can positively affect vision in patients with Leber hereditary optic neuropathy (LHON) in the LEROS study. A clinically relevant benefit is observed in significantly more eyes from patients treated with idebenone compared to those from untreated patients in a historical comparator group.

Yu-Wai-Man et al., 2024, Cell Reports Medicine *5*, 101437
March 19, 2024 © 2024 The Authors.
https://doi.org/10.1016/j.xcrm.2024.101437

Cell Reports Medicine

CellPress
OPEN ACCESS

Article

Therapeutic benefit of idebenone in patients with Leber hereditary optic neuropathy: The LEROS nonrandomized controlled trial

Patrick Yu-Wai-Man,[1,2,3,4,5,38] Valerio Carelli,[6,7,38] Nancy J. Newman,[8] Magda Joana Silva,[9] Aki Linden,[10] Gregory Van Stavern,[11] Jacek P. Szaflik,[12,13] Rudrani Banik,[14] Wojciech Lubiński,[15] Berthold Pemp,[16] Yaping Joyce Liao,[17] Prem S. Subramanian,[18] Marta Misiuk-Hojło,[19] Steven Newman,[20] Lorena Castillo,[21]

(Author list continued on next page)

[1]John van Geest Centre for Brain Repair, University of Cambridge, Cambridge CB2 0PY, UK
[2]MRC Mitochondrial Biology Unit, Department of Clinical Neurosciences, University of Cambridge, Cambridge CB2 0XY, UK
[3]Cambridge Eye Unit, Addenbrooke's Hospital, Cambridge University Hospitals NHS Foundation Trust, Cambridge CB2 0QQ, UK
[4]Moorfields Eye Hospital NHS Foundation Trust, London EC1V 2PD, UK
[5]Institute of Ophthalmology, University College London, London EC1V 9EL, UK
[6]IRCCS Istituto di Scienze Neurologiche di Bologna, Programma di Neurogenetica, 40139 Bologna, Italy
[7]Department of Biomedical and Neuromotor Sciences, University of Bologna, 40127 Bologna, Italy
[8]Departments of Ophthalmology, Neurology, and Neurological Surgery, Emory University School of Medicine, Atlanta, GA 30322, USA
[9]All is Data, 4052 Basel, Switzerland
[10]EstiMates Oy, 20520 Turku, Finland
[11]Washington University in St. Louis, St. Louis, MO 63130, USA
[12]Department of Ophthalmology, Medical University of Warsaw, 02-091 Warsaw, Poland
[13]SPKSO Ophthalmic University Hospital, 00-576 Warsaw, Poland
[14]New York Eye and Ear Infirmary of Mount Sinai, New York, NY 10003, USA
[15]Samodzielny Publiczny Szpital Kliniczny Nr 2 PUM w Szczecinie, 70-111 Szczecin, Poland
[16]Department of Ophthalmology, Medical University of Vienna, 1090 Vienna, Austria
[17]Stanford University Byers Eye Institute, Palo Alto, CA 94303, USA
[18]Sue Anschutz-Rodgers University of Colorado Eye Center, Aurora, CO 80045, USA
[19]Wrocław Medical University, 50-556 Wrocław, Poland
[20]University of Virginia, Charlottesville, VA 22903, USA
[21]Institut Catala de Retina (ICR), 08022 Barcelona, Spain
[22]Department of Ophthalmology, University of Medical Sciences, 60-806 Poznan, Poland
[23]Department of Ophthalmology, Palo Alto Medical Foundation, Palo Alto, CA 94303, USA
[24]Hospital Universitario Ramon y Cajal, IRYCIS, Universidad Alcalá, 28034 Madrid, Spain

(Affiliations continued on next page)

SUMMARY

Leber hereditary optic neuropathy (LHON) is a mitochondrial disease leading to rapid and severe bilateral vision loss. Idebenone has been shown to be effective in stabilizing and restoring vision in patients treated within 1 year of onset of vision loss. The open-label, international, multicenter, natural history-controlled LEROS study (ClinicalTrials.gov NCT02774005) assesses the efficacy and safety of idebenone treatment (900 mg/day) in patients with LHON up to 5 years after symptom onset (N = 199) and over a treatment period of 24 months, compared to an external natural history control cohort (N = 372), matched by time since symptom onset. LEROS meets its primary endpoint and confirms the long-term efficacy of idebenone in the subacute/dynamic and chronic phases; the treatment effect varies depending on disease phase and the causative mtDNA mutation. The findings of the LEROS study will help guide the clinical management of patients with LHON.

INTRODUCTION

Leber hereditary optic neuropathy (LHON) is a rare inherited disease characterized by painless, sequentially bilateral central vision loss.[1–3] Subacute worsening of visual acuity (VA) occurs within 6 months after onset, typically reaching a nadir before entering a dynamic phase, characterized by ongoing changes in the retinal nerve fiber layer and progression of visual field defects.[1] The chronic phase begins after 12 months, by which point VA loss remains severe and permanent in the majority of cases.[1,2,4]

The primary etiological factor for LHON is an mtDNA mutation that affects essential components of complex I (NADH:ubiquinone oxidoreductase).[3] However, the penetrance of LHON is

This is an open access article under the CC BY license (http://creativecommons.org/licenses/by/4.0/).

Jarosław Kocięcki,[22] Marc H. Levin,[23] Francisco Jose Muñoz-Negrete,[24] Ali Yagan,[25] Sylvia Cherninkova,[26] David Katz,[27] Audrey Meunier,[28] Marcela Votruba,[29] Magdalena Korwin,[12,13] Jacek Dziedziak,[12,13,30] Neringa Jurkutė,[4,5,31] Joshua P. Harvey,[4,5] Chiara La Morgia,[6,7,32] Claudia Priglinger,[33] Xavier Llòria,[34] Livia Tomasso,[34] and Thomas Klopstock[35,36,37,39,*] LEROS Study Group

[25]Manchester Royal Eye Hospital, Manchester M13 9WL, UK
[26]UMHAT "Alexandrovska" EAD, 1431 Sofia, Bulgaria
[27]Bethesda Neurology LLC, Bethesda, MD 20852, USA
[28]Department of Ophthalmology, CHU Saint-Pierre, 1000 Brussels, Belgium
[29]Cardiff Eye Unit, University Hospital of Wales, Cardiff CF14 4XW, UK
[30]Department of Experimental and Clinical Physiology, Center for Preclinical Research, Medical University of Warsaw, 02-097 Warsaw, Poland
[31]The National Hospital for Neurology and Neurosurgery, University College London Hospitals NHS Foundation Trust, London WC1N 3BG, UK
[32]IRCCS Istituto delle Scienze Neurologiche di Bologna, 40139 Bologna, Italy
[33]Department of Ophthalmology, University Hospital of the Ludwig-Maximilians-University (LMU), 80336 Munich, Germany
[34]Chiesi Farmaceutici S.p.A., 43100 Parma, Italy
[35]German Center for Neurodegenerative Diseases (DZNE), 81377 Munich, Germany
[36]Munich Cluster for Systems Neurology (SyNergy), 81377 Munich, Germany
[37]Friedrich Baur Institute at the Department of Neurology, LMU University Hospital, LMU Munich, 80336 Munich, Germany
[38]These authors contributed equally
[39]Lead contact
*Correspondence: thomas.klopstock@med.uni-muenchen.de
https://doi.org/10.1016/j.xcrm.2024.101437

incomplete and variable and can therefore not be explained by a single point mutation of mtDNA alone.[5–10] Worldwide, approximately 90% of patients carry one of three common mtDNA mutations, m.11778G>A in *MT-ND4*, m.3460G>A in *MT-ND1*, and m.14484T>C in *MT-ND6*.[2] This proportion can vary by region. For example, in a cohort of over 1,200 Chinese patients, only approximately 40% carried one of these three mutations.[6,9,11] Recessive forms of LHON have recently been described, caused by mutations in the *DNAJC30*, *MCAT*, *MECR*, and *NDUFS2* genes.[12–16]

Therapeutic management of LHON is limited. Several therapeutic approaches are being investigated, including gene therapy treatments, but idebenone is the only currently approved therapy.[17,18] Idebenone is a synthetic short-chain benzoquinone thought to restore mitochondrial function by bypassing the dysfunctional complex I and thus restoring ATP generation,[19,20] and by acting as a potent antioxidant. Recently, additional modes of action have been proposed, including effects on apoptosis, mitophagy, and myelination.[21,22]

In the randomized, double-blind, placebo-controlled Rescue of Hereditary Optic Disease Outpatient Study (RHODOS), patients with LHON and disease onset ≤5 years were treated with idebenone (300 mg 3 times/day) or placebo for 6 months[23] A trend toward improved VA was observed in idebenone-treated patients. In hindsight, the 6-month treatment duration was likely too short to fully capture the potential treatment benefit.

An expanded access program (EAP) allowed for analysis of long-term idebenone treatment in the real world, in subacute/dynamic patients (≤1 year after onset).[24] This noncontrolled study indicated the potential benefit of maintaining idebenone therapy for 24–30 months before classifying patients as nonresponders. This approach resulted in a VA stabilization and/or recovery rate that was higher than expected from limited natural history (NH) studies.[25,26] Based on this cumulative clinical evidence, the European Medicines Agency approved idebenone for the treatment of individuals ≥12 years old with LHON.

Long-term efficacy studies for idebenone in LHON are limited by the lack of direct control data, which are difficult to prospectively compile for rare diseases with an approved treatment. In addition, little data have been collected in the chronic phase (>1 year after onset).[27,28] Here, we present the primary results from LEROS (this study was registered at Clinicaltrials.gov: NCT02774005), an open-label idebenone study in subacute/dynamic and chronic LHON with an external historical control group.

RESULTS

Study populations and baseline characteristics

In LEROS, conducted from 2016 to 2021, 198 of 199 enrolled patients received at least one dose of idebenone (safety population). Of these, postbaseline VA assessments were available for 196 patients (intention to treat [ITT] population). For direct comparisons between the LEROS and comparator groups, only patients with the 3 common mutations were included, resulting in 181 patients in the modified (m)ITT population (Figure S1).

For the NH comparator group, 592 case records were assessed for eligibility, leading to 731 eyes from 372 patients being eligible for matching (Figure S2). Case records of 383 patients were available from the Case Record Survey-1 (CRS-1). Of these, 10 patients had also provided case records in the Case Record Survey-2 (CRS-2) and were excluded from the dataset of CRS-1. Of the remaining 373 case records, 28 were excluded to match the LEROS eligibility criteria, leading to 345 subjects and 690 eyes, respectively (Figure S2). Of these, 358 eyes were excluded due to unknown year of symptom onset, onset of symptoms over 5 years prior or less than 2 VA assessments after onset of symptoms, and previous idebenone use, leading to 168 patients and 332 eyes, respectively, being eligible for

matching to the LEROS group. From CRS-2, there were 219 case records available, 6 of which were excluded due to patients' age of <12 years. From the remaining 213 eligible patients and 426 eyes, 27 eyes were excluded either due to an unknown onset year or having fewer than two VA assessments after onset of symptoms and previous idebenone use. Consequently, 399 eyes from 204 patients were eligible for matching to the LEROS group.

Patient demographics of the ITT population were typical of LHON (Table S4), with a high proportion of males (73.5%) and a mean age at symptom onset of 32.5 years. For efficacy analyses (mITT), eyes were stratified according to disease phase, defined by time since symptom onset at baseline (subacute/dynamic: ≤1 year; chronic: >1 year). Comparison of baseline characteristics of individually matched datasets at each analysis time point (by eyes) (Table 1) revealed important differences in the distribution of mtDNA mutations, gender, age, and VA blindness categories. Other baseline characteristics were comparable, including time since onset of symptoms.

Patient characteristics in the overall NH dataset (N = 592) and in patients eligible for matching (N = 372) are shown in Tables S1 and S2. The proportion of male patients and the distribution of the primary mtDNA mutations was comparable to reports in the literature.[3,29] In patients eligible for matching, the mean follow-up time was 47.9 months (4 years), ranging from 0.1 to 514.1 months (0–42.8 years), the mean time from onset of symptoms to the first visit was 0.4 years, and over 90% of patients had their first visit within 1 year from onset of symptoms (Table S2).

Of the 3,999 VA measurements in the 372 patients eligible for matching in the NH cohort, 51.3% were reported as Snellen fractions (Table S3). The proportion of VA assessments reported as the logarithm of the minimum angle of resolution (logMAR) and decimal scores was balanced with 12.7% and 14.5%, respectively. Approximately 20% of VA measurements recorded an off-chart VA for which no measurement method was available in the case record surveys.

Efficacy

Responder analyses

Subacute/dynamic phase. LEROS met its primary endpoint; in subacute/dynamic eyes the rate of clinically relevant benefit (CRB) from baseline was significantly higher following 12 months of treatment versus matched NH eyes (42.3% [60/142] vs. 20.7% [40/193] [p = 0.002; odds ratio 2.29; 95% confidence limit 1.35–3.88]).

The treatment effect observed in the primary endpoint remained significant in a sensitivity analysis, done through the imputation of missing data, inverse probability of treatment, and an extension of the observation window to 12 ± 3.5 months and 12 ± 4 months. Overall, regardless of the methods used for sensitivity analysis, the estimated difference between the rates of CRB in treated and NH eyes was maintained with a p value between <0.001 and 0.006 in favor of idebenone presenting with odds ratio values between 2.028 and 2.2980 (data not shown).

The observed treatment effect was maintained after 24 months (52.9% vs. 36.0%, p = 0.03) and was primarily driven by a difference in clinically relevant stabilization (CRS) rates between the 2 groups at 12 months (64.5% vs. 22.5%, p < 0.001) and at 24 months (66.7% vs. 46.2%, p = 0.10) (Figure 1A; Tables S5, and S6). Although not statistically significant, the clinically relevant recovery (CRR) rates also indicated a positive treatment effect at 12 months (33.1% vs. 18.1%, p = 0.09) and 24 months (47.9% vs. 33.3%, p = 0.07).

Furthermore, clinically relevant worsening (CRW) rates were significantly lower in treated versus untreated subacute/dynamic eyes at both 12 (29.1% vs. 58.5%, p < 0.001) and 24 months (25.8% vs. 51.0%, p = 0.005). These treatment effects remained significant in a sensitivity analysis with the additional covariates of age at first symptom onset and time since symptom onset (Table S5).

Exploratory stratification of the data into eyes initiating treatment in the subacute (<6 months after onset of symptoms) and in the dynamic phase of the disease (6–12 months after onset of symptoms) confirmed a treatment benefit across all of the responder outcomes (CRB, CRR, CRS, CRW) at 24 months treatment (Figure S3).

Chronic phase. The frequency of a CRB from baseline at 12 months was significantly higher in treated chronic eyes versus matched NH eyes (50.3% vs. 38.6%, p = 0.009). This was maintained at 24 months (49.1% vs. 37.6%, p = 0.02) and was driven by a significantly higher proportion of eyes with a CRR at 12 months (32.9% vs. 19.6%, p = 0.003) and 24 months (31.9% vs. 16.1%, p = 0.001) (Figure 1B; Table S5). CRW rates were significantly lower in treated versus untreated chronic eyes at both time points (at 12 months 4.9% vs. 16.9%, p = 0.006; at 24 months 2.9% vs. 20.0%, p < 0.001).

Impact of LHON-causative mutations. Prespecified subgroup analyses by mtDNA mutation confirmed a therapeutic benefit in eyes with the m.11778G>A mutation (Figure 1; Table S7). In the subacute/dynamic phase, treatment significantly increased the rate of CRB, CRR, and CRS, and significantly reduced CRW compared to matched NH eyes at 12 and 24 months. In the chronic phase, a significant treatment benefit was found for CRB and CRW at both time points.

In the LEROS study, eyes with the m.3460G>A mutation did not benefit from idebenone treatment, regardless of disease phase. At 24 months, CRB and CRR were nonsignificantly lower, and CRW was significantly higher in treated subacute/dynamic eyes versus the NH control. In the chronic phase, differences were less pronounced, nonsignificant, and reversed for CRR and CRW compared to subacute/dynamic eyes.

In treated subacute/dynamic eyes with the m.14484T>C mutation, CRB, CRR, and CRS rates were comparable to the NH cohort at 24 months, and a nonsignificant reduction in CRW was observed. Significantly increased CRB and CRR rates were observed in treated, chronic eyes at 24 months.

The ratio of VA recovery versus worsening at 24 months (from baseline) in each subpopulation is highlighted when displayed as sigmoid plots (Figure 2).

Impact of age and gender on responder analyses. Subgroup analyses of CRB stratified by age at symptom onset showed a significant treatment benefit in eyes of patients ≥18 years at onset, both for subacute/dynamic and chronic eyes. Subgroup analyses of CRB by gender demonstrated a significant treatment benefit in the eyes of females with chronic LHON, whereas the benefit did not reach significance in males (Figure S4).

Table 1. Demographic and baseline characteristics for eyes with 12- and 24-month VA assessments, by disease phase (mITT)

	Subacute/dynamic eyes				Chronic eyes			
	12 months		24 months		12 months		24 months	
	Idebenone	NH	Idebenone	NH	Idebenone	NH	Idebenone	NH
Characteristic	(N = 142)	(N = 193)	(N = 121)	(N = 75)	(N = 143)	(N = 153)	(N = 116)	(N = 93)
mtDNA mutations, n (%)								
m.11778G>A	68 (47.9)	138 (71.5)	60 (49.6)	47 (62.7)	105 (73.4)	102 (66.7)	82 (70.7)	51 (54.8)
m.3460G>A	32 (22.5)	34 (17.6)	26 (21.5)	18 (24.0)	23 (16.1)	26 (17.0)	23 (19.8)	24 (25.8)
m.14484T>C	42 (29.6)	21 (10.9)	35 (28.9)	10 (13.3)	15 (10.5)	25 (16.3)	11 (9.5)	18 (19.4)
Gender, n (%)								
Male	98 (69.0)	162 (83.9)	84 (69.4)	64 (85.3)	113 (79.0)	119 (77.8)	96 (82.8)	75 (80.6)
Female	44 (31.0)	31 (16.1)	37 (30.6)	11 (14.7)	30 (21.0)	34 (22.2)	20 (17.2)	18 (19.4)
Age at first symptom onset, year								
Mean ± SD	31.4 ± 14.3	31.7 ± 14.5	31.9 ± 14.1	31.2 ± 15.9	32.6 ± 15.8	29.0 ± 15.6	32.9 ± 16.2	25.5 ± 14.0
Min–max	12.1–78.2	13.0–75.0	12.1–78.2	11.0[a] – 63.0	8.8[b]–78.2	7.0[c]– 63.0	8.8[b]–78.2	7.0[d]–63.0
Age at first symptom onset, by gender, year, mean ± SD								
Female	37.6 ± 14.0	37.8 ± 13.0	36.3 ± 13.3	46.0 ± 10.8	32.6 ± 20.4	37.1 ± 15.7	35.3 ± 21.8	30.7 ± 13.7
Male	28.6 ± 13.6	30.5 ± 14.5	30.0 ± 14.2	28.7 ± 15.3	32.5 ± 14.5	26.6 ± 14.8	32.3 ± 14.9	24.3 ± 13.8
Age, year								
Mean ± SD	32.0 ± 14.3	32.0 ± 14.5	32.5 ± 14.2	31.7 ± 15.9	35.1 ± 15.8	31.4 ± 15.6	35.3 ± 16.1	28.1 ± 13.6
Min–max	12.6–79.2	13.0–75.2	12.6–79.2	11.7–63.9	12.1–79.2	11.1–66.8	12.1–79.2	11.5–64.3
Age, n (%)								
<18 yr	22 (15.5)	9 (4.7)	20 (16.5)	8 (10.7)	20 (14.0)	24 (15.7)	16 (13.8)	16 (17.2)
≥18 yr	120 (84.5)	184 (95.3)	101 (83.5)	67 (89.3)	123 (86.0)	129 (84.3)	100 (86.2)	77 (82.8)
Months since onset								
Mean ± SD	6.03 ± 3.08	4.65 ± 2.84	6.01 ± 3.12	5.17 ± 3.06	29.29 ± 14.30	28.60 ± 13.07	29.20 ± 13.94	30.55 ± 14.02
Min–max	0.00–11.73	0.00–10.87	0.00–11.73	0.00–10.91	12.02–58.32	12.02–58.18	12.02–58.32	12.55–59.79
VA, logMAR								
Mean ± SD	1.28 ± 0.48	1.26 ± 0.52	1.26 ± 0.47	1.31 ± 0.49	1.33 ± 0.53	1.25 ± 0.63	1.32 ± 0.53	1.24 ± 0.61
Min–max	−0.12 to 1.80	−0.10 to 1.80	−0.12 to 1.80	0.00–1.80	−0.04 to 1.80	−0.11 to 1.80	0.06–1.80	0.00–1.80
Blindness category (%)								
Off-chart	25 (17.6)	63 (32.6)	18 (14.9)	26 (34.7)	47 (32.9)	64 (41.8)	36 (31.0)	33 (35.5)
1.00 ≤ logMAR ≤1.68	86 (60.6)	90 (46.6)	76 (62.8)	36 (48.0)	62 (43.4)	51 (33.3)	52 (44.8)	37 (39.8)
logMAR <1.00	31 (21.8)	40 (20.7)	27 (22.3)	13 (17.3)	34 (23.8)	38 (24.8)	28 (24.1)	23 (24.7)

Max, maximum; min, minimum. See also Tables S1, S2, S4, and S9.
[a]2 eyes from patients <12 years (both 11 years).
[b]8 eyes from patients <12 years, thereof 2 eyes <9 years.
[c]21 eyes from patients <12 years, thereof 4 eyes <9 years.
[d]18 eyes from patients <12 years, thereof 4 eyes <9 years.

Impact of treatment duration on CRR probability

In the eyes of subacute/dynamic patients from the LEROS ITT population (N = 195), the Kaplan-Meier estimate of a first CRR from baseline increased progressively from 18.4% at month 6 to 34.9% at month 12, reaching 47.3% at month 24 (Figure 3A). In the chronic phase (N = 186), the incidence of a first CRR from baseline increased progressively from 18.2% at month 6 to 26.7% at month 12, reaching 29.1% by month 24 (Figure 3B).

Magnitude of VA change

To assess the impact of treatment on the magnitude of VA change in treated eyes versus the NH cohort, the differences in VA change (least squares [LS]-means) from baseline to months 12 and 24 were compared (mITT vs. NH) (Figure 4; Table S8).

CellPress
OPEN ACCESS

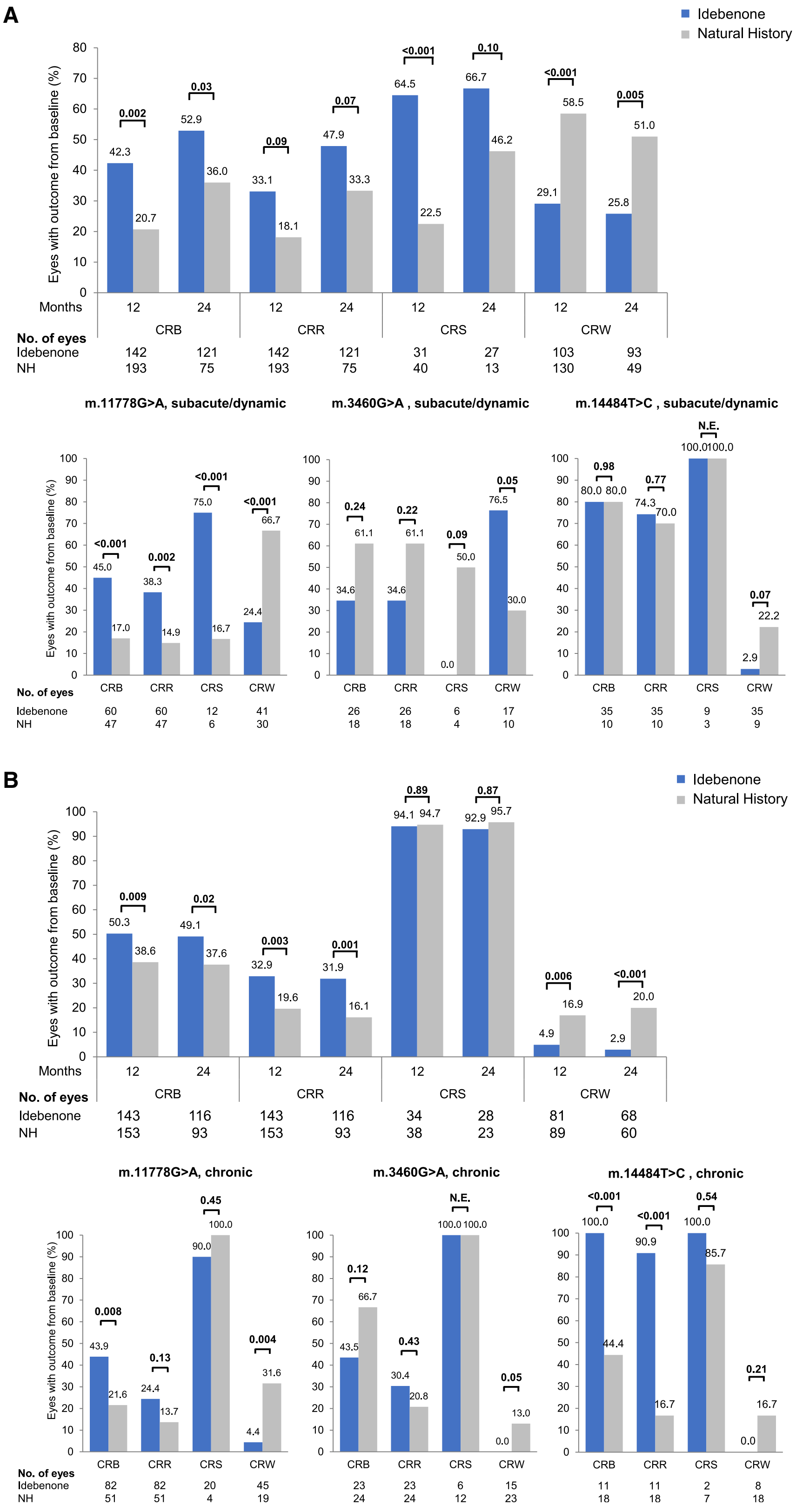

Figure 1. Responder analyses

Responder analyses in (A) subacute/dynamic eyes and (B) chronic eyes, overall and by mutation (mITT vs. matched NH). For CRS analyses, only eyes with a baseline VA <1.0 logMAR were included. Only eyes with a baseline VA ≤1.68 logMAR (on-chart VA) were included in analyses of CRW. Brackets indicate p values. See also Figures S3 and S4 and Tables S5–S7. N.E., not estimable.

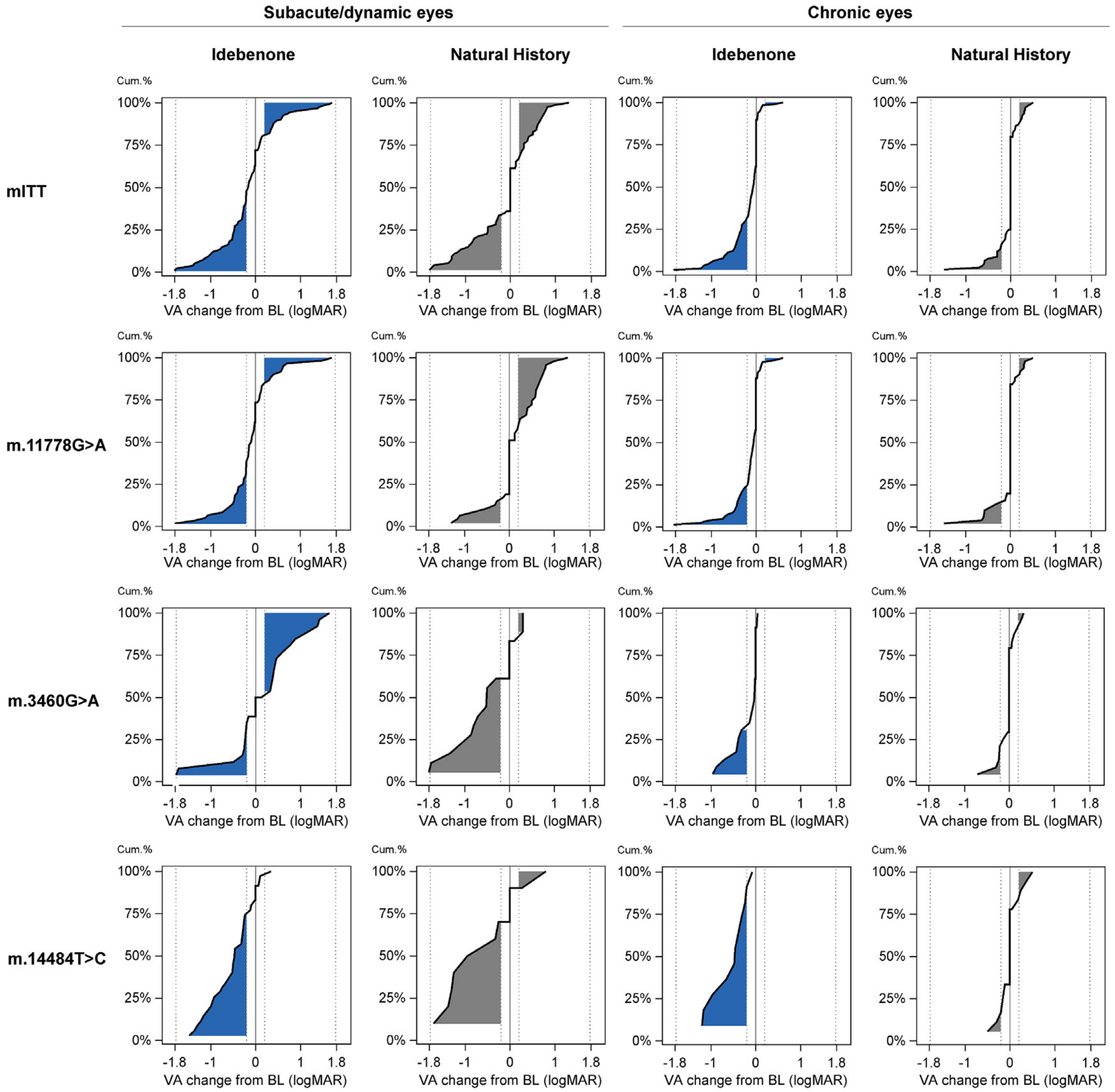

Figure 2. Cumulative frequency of change in VA from baseline to 24 months by disease phase and mutation status (mITT vs. matched NH)

The dotted lines at −0.2 logMAR and +0.2 logMAR indicate the thresholds for improvement and worsening, respectively, by at least 10 letters on the ETDRS chart. BL, baseline; cum, cumulative.

In subacute/dynamic eyes, VA improved from 1.29 logMAR at baseline to 1.20 logMAR at 12 months in treated eyes and worsened from 1.26 to 1.32 logMAR in the matched NH group. The difference in magnitude of VA change between treated eyes and the NH group correspond to a significant relative improvement of −0.12 logMAR in favor of idebenone ($p = 0.03$), equivalent to an improvement of 6 letters on the Early Treatment Diabetic Retinopathy Study (ETDRS) chart. At 24 months, there was a nonsignificant improvement in treated eyes of −0.03 logMAR relative to the control group.

Subanalyses by mutation showed a consistent treatment benefit over time in subacute/dynamic eyes with the m.11778G>A mutation. At 12 months, treated eyes improved from 1.29 logMAR at baseline to 1.25 logMAR, whereas matched NH eyes worsened from 1.27 logMAR to 1.57 logMAR, corresponding to a relative improvement in treated eyes of −0.33 logMAR ($p < 0.001$) (16 ETDRS letters) in favor of idebenone. This was comparable at 24 months, with an improvement from 1.31 logMAR at baseline to 1.22 logMAR in treated eyes versus a worsening from 1.36 logMAR to 1.53 logMAR in the matched control group, corresponding to a relative improvement of −0.32 logMAR ($p = 0.002$) (16 ETDRS letters) in favor of idebenone.

In subacute/dynamic m.3460G>A and m.14484T>C eyes, differences in VA change from baseline between the treatment groups were nonsignificant at both time points, except for eyes with the m.3460G>A mutation at 24 months. Here, treated eyes worsened from 1.29 logMAR at baseline to 1.48 logMAR, whereas the matched NH group improved from 1.45 logMAR at baseline to 0.95 logMAR, equivalent to a relative worsening

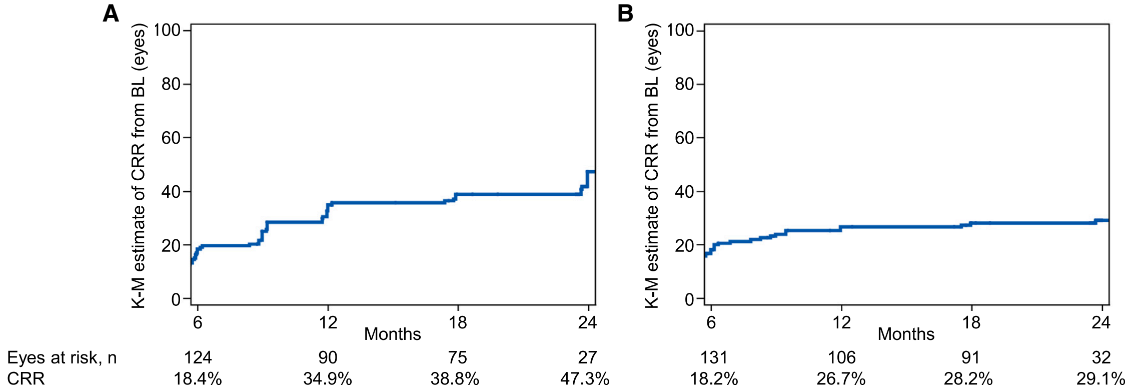

Figure 3. Kaplan-Meier (K-M) analysis

Kaplan-Meier analysis of initial CRR from baseline up to month 24 as a function of treatment duration in (A) subacute/dynamic and (B) chronic eyes (ITT). CRR is presented as the Kaplan-Meier estimate.

in treated versus untreated eyes of 0.53 logMAR ($p = 0.001$) (26 ETDRS letters).

In the chronic phase, a significant treatment benefit was observed at both analysis time points for the overall mITT population and the m.11778G>A mutation, and for the m.14484T>C mutation at 24 months. In the overall population, VA improved from 1.16 logMAR at baseline to 1.11 logMAR at 12 months in treated eyes, versus a worsening from 1.11 logMAR to 1.21 logMAR in the control group, corresponding to a relative improvement of −0.10 logMAR ($p = 0.004$) (5 ETDRS letters) in favor of idebenone. This was similar at 24 months, with an improvement from 1.20 logMAR to 1.07 logMAR in treated eyes versus a worsening from 1.18 logMAR to 1.24 logMAR in the control group, corresponding to a relative improvement of −0.17 logMAR ($p < 0.001$) (8 ETDRS letters).

In m.11778G>A eyes, treatment improved VA from 1.35 logMAR at baseline to 1.17 logMAR at 12 months, versus an improvement in the control group from 1.49 logMAR to 1.27 logMAR, corresponding to a relative improvement of −0.10 logMAR ($p = 0.02$) (5 ETDRS letters) in favor of idebenone. This was comparable at 24 months, with an improvement from 1.34 logMAR at baseline to 1.15 logMAR in treated eyes, versus an improvement from 1.54 logMAR to 1.26 logMAR in the control group, corresponding to a relative improvement of −0.11 logMAR ($p = 0.04$) (5 ETDRS letters) in favor of idebenone.

In chronic m.3460G>A eyes, no statistically significant differences were found between treated eyes and the NH group at 12 or 24 months.

Treated chronic m.14484T>C eyes improved from 1.21 logMAR at baseline to 0.75 logMAR at 24 months, whereas the control group worsened from 1.00 logMAR to 1.27 logMAR, corresponding to a relative improvement of −0.52 logMAR ($p < 0.001$) (26 ETDRS letters) in favor of idebenone. A similar trend was observed at 12 months, but it was not statistically significant.

To better understand the behavior of m.3460G>A eyes, we displayed the VA score of each eye at baseline versus the VA score at the corresponding visit time (Figure S5). The resulting scatterplots confirm the findings above. In addition, they show that many subacute/dynamic eyes with the m.3460G>A mutation worsened to off-chart VA after 24 months of treatment, with only 2 eyes improving considerably. This is opposed to the matched NH group, in which 5 eyes improved to <0.5 logMAR, and few eyes worsened to off-chart VA.

Safety

The NH populations did not include any safety data. The LEROS safety population included all of the patients enrolled in LEROS, who received at least one dose of study treatment (N = 198). Baseline characteristics are summarized in Table S9. The distribution of gender and mutation status, as well as mean age, were typical of patients with LHON.

The mean duration of treatment in the safety population was 589.17 days (range: 1–806 days). The majority of treatment-emergent adverse events (TEAEs) were considered to be of mild to moderate intensity, with only 11.3% considered to be related to idebenone treatment by the investigator, during a mean duration of treatment of 247 days (Table S10). By System Organ Class, the majority of treatment-related TEAEs were classified as investigations (36 events), followed by gastrointestinal disorders (28 events); nervous system disorders (9 events); general disorders and administration site conditions (6 events); renal and urinary disorders (6 events); psychiatric disorders (4 events); eye disorders (3 events); 2 events each in musculoskeletal and connective tissue disorders, respiratory, thoracic, and mediastinal disorders, and skin and subcutaneous tissue disorders; and 1 event each in infections and infestations, metabolism and nutrition disorders, and vascular disorders.

A total of 154 patients (77.8%) received treatment for >12 months and 106 (53.5%) for 24 months, and 154 (77.8%) patients reported TEAEs.

The frequency and type of TEAEs were as expected and no new safety signals were observed. Overall, 891 TEAEs were observed. A total of 13 (6.6%) patients reported severe TEAEs, and 49 (24.7%) patients reported TEAEs that were considered by the investigator to be treatment related. Ten (5.1%) had AEs that led to permanent discontinuation of study treatment. Twenty-seven (13.6%) patients experienced serious AEs. One TEAE led to death (alcoholic liver failure) and was deemed

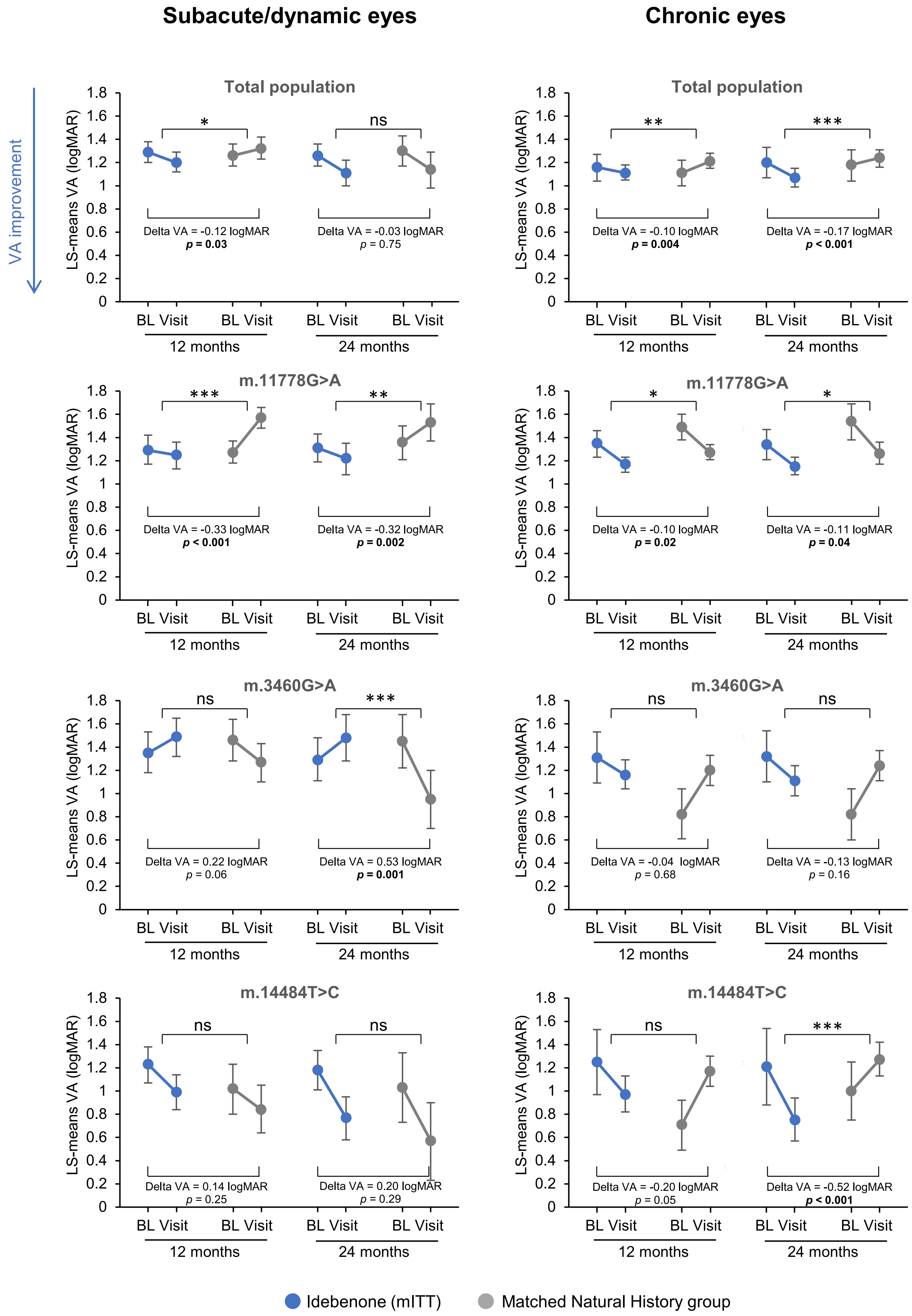

(legend on next page)

unrelated to study treatment by both the investigator and sponsor. The most frequent TEAEs were headache and nasopharyngitis (Table 2).

Four (0.4%) mild events of increased blood cholesterol levels were observed in 4 (2.0%) patients and 6 (0.7%) mild events of increased triglyceride levels were observed in 5 (2.5%) patients. One event of increased liver function test was observed in 1 treated patient (1/198; 0.5%) across the study period. This event was of moderate severity and deemed not related to study treatment by the investigator.

DISCUSSION

The results of LEROS substantially contribute to the existing body of evidence, suggesting a benefit of idebenone treatment in LHON, and at least partially address the main limitations of previous studies: the short duration (6 months) of the only placebo-controlled randomized trial (RHODOS),[23] and the lack of a control group in other studies.[24,27,28,30,31] In addition, LEROS provides supportive efficacy data for the chronic disease phase, a group of patients who, until recently, have been underrepresented.[27,28,30]

Baseline demographics for LEROS mITT and matched NH groups were typical of LHON. Differences in the distribution of mtDNA mutations, gender, and age have been considered when interpreting the results as discussed further below. There were also some differences in the distribution of eyes among VA categories (Table 1), in particular, there was a shift from the legally blind category toward the off-chart category in the matched NH groups compared to the corresponding mITT groups. Potential explanations for these differences are considered in the limitations section below.

Efficacy of idebenone in subacute/dynamic LHON

Idebenone treatment provided a therapeutic benefit in the subacute/dynamic phase when compared to the external NH group. This was observed as an increased proportion of eyes experiencing a CRB when treated, which was largely driven by an increased rate of CRS (Figure 1A; Table S5). Treatment also reduced the rate of CRW.

The CRB rate for treated eyes at 12 months (42.3%) was similar to that from a post hoc analysis of RHODOS at 6 months (39.6% of patients) (data not shown). Despite CRR from baseline likely being lower compared to CRR from nadir, the CRR rate from baseline at 24 months (47.9%) was consistent with real-world values of CRR from nadir reported in patients treated with idebenone: 46.0% in the EAP[24] and 53% in a retrospective Dutch cohort study.[31]

The CRS rate (66.7% at 24 months), based on eligible eyes with baseline VA <1.0 logMAR, was higher compared to those reported previously (22% in the Dutch cohort study and 50% in the EAP).[24,31] The reasons for these differences are likely multifactorial and include the distribution of mutations, age at onset of symptoms, duration of symptomatic disease, and VA at baseline.

Because there were differences in the distribution of the three mtDNA mutations between the mITT and NH groups, we performed subgroup analyses to understand the impact of bias. These subanalyses offer useful insights that complement the combined group data, with the caveat that the number of eyes, and therefore, the statistical power, is reduced. This was especially the case for the m.3460G>A and m.14484T>C subgroups. Our findings substantiate previous evidence indicating that idebenone treatment response varies according to the underlying causative mtDNA mutation.[23,24]

A significant therapeutic benefit was observed in all four VA response measures in subacute/dynamic m.11778G>A eyes (Figures 1A and 2; Table S7). These results are noteworthy considering the poor visual prognosis with this mutation.[25,32] The m.14484T>C mutation confers a relatively favorable prognosis[33,34] that was also observed in LEROS as high rates of spontaneous recovery (and stabilization) in the subacute/dynamic phase. This likely masked a relative treatment benefit in these eyes, as previously observed in RHODOS.[23]

In subacute/dynamic m.3460G>A eyes, treatment significantly increased the proportion of eyes with a CRW, and resulted in nonsignificant, negative trends for CRR, CRS, and CRB. Little previous data are available to assess the efficacy of idebenone in patients carrying the m.3460G>A mutation. A breakdown of results by mutation was not carried out in RHODOS.[23] In the EAP, a CRR rate of 41% was observed in treated patients with the m.3460G>A mutation. This is roughly comparable to the rate in LEROS at 24 months (34.6%).

Although spontaneous recovery of vision in NH eyes carrying the m.11778G>A or m.14484T>C mutation was largely consistent with previous data,[3,5,25,34,35] it was more frequent in subacute/dynamic (but not chronic) m.3460G>A eyes (61.1% at 24 months vs. 15%–25% in previous reports).[3,5,34–37] Spontaneous recovery may be overrepresented in the NH cohort and could partly explain the apparent trend toward a negative treatment effect. It is feasible that this potentially “milder” disease course could also manifest as a reduced rate of CRW; however, this cannot be verified due to the lack of comparable data in the literature. It is also important to note the relatively small sample size used for assessing outcomes in subacute/dynamic m.3460G>A eyes, which was considerably smaller than for the other two LHON mtDNA mutations. The rates of CRW in subacute/dynamic eyes with the m.3460G>A mutation should, therefore, be interpreted with caution.

It is also possible that the response to idebenone is muted to a degree in the LEROS m.3460G>A subgroup. Low NQO1 protein levels hamper the reduction of idebenone from its oxidized form, which is a precondition for its therapeutic effect, and this has recently been associated with idebenone cytotoxicity.[38,39] The m.3460G>A mutation has recently been reported to be particularly sensitive to NQO1 protein levels, greatly limiting the therapeutic efficacy of idebenone.[40] A hypothesis that may partially explain the observations in the mITT population is that a subgroup of patients

Figure 4. Change in VA (LS-mean VA) from baseline to 12 and 24 months (mITT vs. matched NH)
Difference in change of LS-mean VA from baseline to visit time point between treated eyes and matched eyes in the NH group (delta VA) were calculated using analysis of covariance (ANCOVA) with fixed factors of treatment, gender, mutation, and VA at baseline as a covariate. Error bars indicate 95% confidence limits. ns $p > 0.05$; *$p \leq 0.05$; **$p \leq 0.01$; ***$p \leq 0.001$. See also Figure S5 and Table S8. ns, nonsignificant.

Table 2. TEAEs (MedDRA preferred term) occurring in >5% of patients

Preferred term	TEAE f (%) (F = 891)	Patients n (%) (N = 198)	Days in treatment Mean (SD)	Days in treatment Min–max
Headache	131 (14.7)	37 (18.7)	259.0 (230.3)	1.0–760.0
Nasopharyngitis	51 (5.7)	33 (16.7)	217.2 (213.5)	1.0–714.0
Diarrhea	28 (3.1)	19 (9.6)	142.8 (212.0)	1.0–705.0
Alanine aminotransferase increased	18 (2.0)	17 (8.6)	267.8 (239.2)	19.0–734.0
Blood creatine phosphokinase increased	17 (1.9)	15 (7.6)	219.8 (226.3)	22.0–734.0
Nausea	20 (2.2)	15 (7.6)	135.2 (183.3)	1.0–714.0
Aspartate aminotransferase increased	14 (1.6)	14 (7.1)	276.8 (239.2)	33.0–734.0
Oropharyngeal pain	22 (2.5)	14 (7.1)	258.5 (203.1)	4.0–718.0
Abdominal pain upper	14 (1.6)	13 (6.6)	80.4 (126.9)	1.0–479.0
Cough	14 (1.6)	12 (6.1)	232.6 (218.6)	4.0–698.0
γ-Glutamyl transferase increased	10 (1.1)	10 (5.1)	246.3 (211.2)	19.0–561.0

MedDRA, Medical Dictionary for Regulatory Activities. See also Table S10.

with the m.3460G>A mutation carry specific polymorphisms in homozygous or compound heterozygous combinations in the *NQO1* gene (encoding the NAD[P]H:quinone oxidoreductase), which are known to affect the amount of NQO1 protein.[41–43] This hypothesis needs to be investigated as part of future work, for example, by stratification of the data by NQO1 pharmacogenetics.

Subgroup analyses by age at symptom onset demonstrated a significantly higher rate of CRB at 24 months in treated versus untreated eyes of patients ≥18 years regardless of disease phase, consistent with the overall cohort (Figure S4). In the eyes of patients aged <18 years at onset, CRB rates were similar between treated and NH groups, partially because spontaneous recovery was comparatively high in the NH population. This may be explained by the high proportion of eyes with the m.3460G>A mutation in the NH group (8/22) compared to the treated group (4/22) (data not shown). As mentioned above, the rate of spontaneous CRR was unexpectedly high for this mutation and likely contributed to the high rate of CRB in the <18 years NH group. Furthermore, childhood-onset LHON has a relatively good visual prognosis, with a high rate of spontaneous visual recovery for the m.3460G>A mutation.[44,45] Notably, none of the treated eyes and only 2/75 eyes in the subacute/dynamic NH group at 24 months were of patients <12 years old at symptom onset; both were 11 years old.

Subgroup analyses of CRB by gender revealed no significant differences in CRB rates between treated eyes and the matched NH cohort, except in chronic eyes of female patients (Figure S3).

The Kaplan-Meier estimates of CRR at 12 and 24 months (Figure 3) compared well to outcomes of responder analyses, both in the subacute/dynamic and chronic phase (Figure 1). In the subacute/dynamic phase, the probability of a CRR increased from 34.6% at 12 months to 47.3% at 24 months, comparable to data from the EAP,[24] where longer treatment duration increased the likelihood of eyes achieving CRR, reaching 44.4% at 24 months[24] Similarly, a retrospective Dutch cohort study showed a substantial increase in CRR rates between 12 and 24 months of idebenone treatment.[31] Together, these data indicate that a considerable proportion of patients benefit from treatment beyond 12 months, in particular those in the subacute/dynamic phase.

When considering absolute VA change from baseline to month 24, no significant treatment benefit was observed in the overall subacute/dynamic cohort. This is not entirely surprising when accounting for the influence of the m.3460G>A subgroup described above. When stratified according to the causative mtDNA mutation, a treatment benefit became apparent in the m.11778G>A subacute/dynamic population, for which treatment improved VA by 16 ETDRS letters relative to the NH group.

Efficacy of idebenone in chronic LHON

Idebenone treatment provided a benefit in chronic LHON with a significantly increased CRB rate at 12 and 24 months, driven by CRR (Figure 1B; Table S5). An RHODOS post hoc analysis revealed a CRR rate of 23.5% in chronic eyes after 6 months of treatment.[46] In LEROS, the CRR rate was a comparable 25.5% at month 6 (data not shown) and increased to 31.9% at month 24, suggesting a higher benefit with prolonged treatment. The rate of spontaneous CRR in the RHODOS placebo arm was 5.3%, lower than the 15.2% in the LEROS NH group (month 6, data not shown), or at any other time point.

CRS is not a valid comparative outcome measure in the chronic phase. In mild cases, in which VA remains <1.0 logMAR beyond 1 year after onset, it is likely to remain in this range regardless of treatment. This was indeed the case in LEROS, with similarly high CRS rates (>90%) in both groups. The same would have been expected for CRW, but the rate was higher than expected (up to 20%) in the NH group and was significantly reduced by idebenone treatment. This result is interesting because it suggests the potential for further worsening in the chronic phase; treatment could therefore have a positive impact in this regard and warrants further study.

Subgroup analyses based on mtDNA mutation showed a significant therapeutic benefit in chronic m.11778G>A and m.14484T>C eyes (Figure 1B; Table S7). At 24 months, idebenone significantly increased the CRB rate and reduced the CRW rate in m.11778G>A eyes and improved the mean VA by 5 ETDRS letters relative to the NH group.

In subacute/dynamic m.14484T>C eyes, the outcomes were generally favorable, even in the absence of treatment, as expected based on the greater likelihood of spontaneous visual recovery with this mutation.[33,34] This was not the case in the chronic phase, in which a significant treatment benefit was observed with improved CRB and CRR rates at month 24, and a relative mean VA improvement of 26 ETDRS letters in favor of idebenone.

In chronic m.3460G>A eyes, idebenone treatment appeared to have little impact overall, but the clear negative trend against treatment was not apparent as for the subacute/dynamic phase. As already mentioned, a degree of caution is needed in interpreting these observations given the small number of eyes and the unexpectedly mild disease severity in the NH group.

Subanalyses by age at symptom onset revealed a comparable CRB increase with idebenone treatment in the ≥18 years group as observed for the mITT, and a similar, albeit nonsignificant, trend in the group <18 years (Figure S4). In the chronic phase at month 24, 8/116 eyes belonged to idebenone-treated patients aged <12 years old at symptom onset, and of those, 2 were from a patient <9 years old (8.8 years). In the corresponding NH group, 18/93 eyes were from patients <12 years old at symptom onset, with 4 from patients <9 years old. A recent study in 68 patients with childhood-onset LHON reported a better visual outcome in patients <9 years old at symptom onset compared to those aged 9–12 years old, who showed more similarity with the classical adult form of LHON.[47] The low number of patients aged <9 years at symptom onset in both groups is, therefore, unlikely to have had a strong impact on the analyses of the overall populations and by mutation.

When assessing the impact of gender, idebenone particularly affected the CRB rate in females with chronic LHON. Anecdotal evidence suggests female LHON patients worsen after menopause (unpublished data).[48–50] This may result in a second "peak" in the chronic phase.

As in the subacute/dynamic phase, and in prior studies,[24,27,28] longer treatment duration increased the likelihood of CRR in the chronic phase (Figure 3). This increase was largely observed up to month 12, after which further increase was relatively small (reaching 29% at month 24). Idebenone significantly improved the mean VA change from baseline at 24 months in the mITT population compared to the matched NH cohort. This was driven by a significant treatment benefit in both m.11778G>A and m.14484T>C eyes, in which VA in the NH cohort remained nearly unchanged at 24 months versus baseline. However, treatment significantly improved VA, particularly for the m.14484T>C mutation.

Safety

The safety profile in idebenone-treated patients was similar to that from previous studies in regard to the type and frequency of treatment-emergent adverse events (Tables 2, S8, and S9).[23,24]

Conclusions

LEROS confirmed the benefit of idebenone in LHON, including in the chronic phase (1–5 years since onset). Increased treatment duration to 24 months maximized the rate of VA recovery. The treatment effect varied depending on disease phase and the causative mtDNA mutation, with a consistent treatment benefit observed for patients with the most common m.11778G>A mutation regardless of disease phase, and for patients with the m.14484T>C mutation in the chronic phase. Further study of idebenone use in patients carrying the m.3460G>A mutation is needed to clarify treatment benefits. In the meantime, patients carrying this particular mtDNA mutation who are in the subacute/dynamic phase should be adequately counseled to allow them to make an informed decision as to whether treatment with idebenone should be initiated.

Limitations of the study

Although desirable, another placebo-controlled trial after RHODOS would be logistically almost impossible to conduct considering the availability of idebenone as an approved treatment in several countries and the relative rarity of LHON. Use of an external historical control group is the best approximation, but it comes with several limitations, such as lack of standardized VA measurements, potential for missing data points, and inconsistent follow-up.[25,51] For example, it has been shown that patients tend to perform better on ETDRS charts than Snellen charts.[52] This may explain, in part, the higher proportion of eyes with off-chart VA at baseline in the NH group, since Snellen charts were used in the NH group in some cases, but not in LEROS (Table S3). It is unclear to what extent this may have influenced outcomes. On the one hand, a high proportion of eyes with off-chart VA at baseline could give more room for improvement; on the other hand, it could indicate that a larger proportion of NH eyes had already deteriorated beyond the point of therapeutic rescue. Considering that for any one patient, the same methods would typically be used over time, their influence on outcomes compared from baseline are likely minimal. Finally, the difference in rates of the various responder analysis outcomes were sufficiently high in many cases, making it unlikely that they are primarily caused by systematic differences in VA assessment method. The present study contains data from patients dating back to the 1950s and we cannot rule out or confirm secular trends in VA outcomes in the NH control population. Nevertheless, over half of eligible eyes in the NH population were derived from CRS-2 (399/731 eyes; see Figure S2), which included case records from 2000 and later.

Finally, the need to conduct subanalyses resulted in a low number of eyes for some comparisons, limiting data robustness. This study was powered for the primary endpoint and the difference in outcomes between subgroups should not be overinterpreted. Although stringent matching criteria were applied for comparison to LEROS data, it is not possible to fully exclude biases, particularly in mutational subgroup analyses involving fewer eyes. We also did not correct the data for the interdependence of eyes to reduce complexities.

STAR★METHODS

Detailed methods are provided in the online version of this paper and include the following:

- KEY RESOURCES TABLE
- RESOURCE AVAILABILITY

- Lead contact
- Materials availability
- Data and code availability
- EXPERIMENTAL MODEL AND SUBJECT DETAILS
- METHOD DETAILS
  - Study design and objectives
  - Sample size determination
  - Eligibility criteria and study populations
  - Natural history comparator population
- QUANTIFICATION AND STATISTICAL ANALYSIS
  - Definition of outcome measures
  - Conversion of VA measurements to logMAR values
  - Statistical methods
- ADDITIONAL RESOURCES

SUPPLEMENTAL INFORMATION

Supplemental information can be found online at https://doi.org/10.1016/j.xcrm.2024.101437.

ACKNOWLEDGMENTS

The authors express their gratitude to all nonauthor study investigators, sites, and participating patients. In particular, the authors thank the following collaborators for their contribution in recruiting patients and collecting data: Cécile Andris, C.H.U. Sart Tilman, Liege, Belgium; Antonella Boschi, Cliniques Universitaire Saint-Luc, Brussels, Belgium; Ingele Casteels, UZ Leuven-Campus Sint-Rafaël, Leuven, Belgium; Bart Leroy, Universitair Ziekenhuis Gent, Gent, Belgium; Sergio Silva, Centro Hospitalar de São João, EPE, Porto, Portugal; Ayman Khaier, Queen's Hospital, Romford, UK; Derek Kunimoto, Retinal Consultants of Arizona, Phoenix, Arizona, USA.

P.Y.-W.-M. is supported by an Advanced Fellowship Award (NIHR301696) from the UK National Institute for Health Research (NIHR); he also receives funding from Fight for Sight (UK), the Isaac Newton Trust (UK), Moorfields Eye Charity (GR001376), the Addenbrooke's Charitable Trust, Cambridge University Hospitals, the National Eye Research Centre (UK), the International Foundation for Optic Nerve Disease (IFOND), the NIHR as part of the Rare Diseases Translational Research Collaboration, the NIHR Cambridge Biomedical Research Centre (NIHR203312), and the NIHR Biomedical Research Centre based at Moorfields Eye Hospital NHS Foundation Trust and UCL Institute of Ophthalmology (NIHR203322). The views expressed are those of the author(s) and not necessarily those of the National Health Service, the NIHR or the Department of Health. V.C. is supported by grants from the Italian Ministry of Health (RF-2018-12366703), the Italian Ministry of University and Research (20172T2MHH), and Telethon-Italy (GGP20115); patient organizations MITOCON and IFOND; and patients' donations. T.K. acknowledges support by the German Federal Ministry of Education and Research (BMBF, Bonn, Germany) through grants to the German Network for Mitochondrial Disorders (mitoNET, 01GM1906A) and to the E-Rare project GENOMIT (01GM1920B).

This study was funded by Santhera Pharmaceuticals, Pratteln, Switzerland. Medical writing support was provided by Marie Kirsten from nspm ltd, Switzerland, and financially supported by Chiesi Farmaceutici S.p.A., Parma, Italy.

AUTHOR CONTRIBUTIONS

M.J.S. and A.L. conducted the statistical analysis of the data. T.K., X.L., and M.J.S. significantly contributed to the design of the study. P.Y.-W.-M., V.C., T.K., and L.T. made significant contributions to the writing of this manuscript. All of the other authors contributed patients to the study and critically reviewed the manuscript.

DECLARATION OF INTERESTS

P.Y.-W.-M., V.C., N.J.N., B.P., L.C., and T.K. received research support and/or personal compensation from Santhera Pharmaceuticals, Chiesi, and GenSight Biologics. P.Y.-W.-M. is also a consultant for Neurophth and Stoke Therapeutics. N.J.N. is also a consultant for Santhera Pharmaceutical, Chiesi, GenSight Biologics, Stoke Therapeutics, Avidity Biosciences, and Neurophoenix SAS, and has been a speaker and contributing writer for educational activities under the auspices of WebMD and First Class.

P.S.S. is a consultant to GenSight Biologics and received research support from GenSight Biologics and Santhera Pharmaceuticals. M.H.L. received personal compensation from Santhera Pharmaceuticals. C.L.M. has acted as a consultant for Chiesi Farmaceutici, Regulatory PharmaNet, and Thenewway srl, and has also received speaker honoraria and/or financial support for meetings from these companies, as well as from First Class srl and Biologix. C.L.M. has also acted as a principal or study investigator for clinical trials sponsored by GenSight Biologics, Santhera Pharmaceuticals, Stoke Therapeutics, and Reneo Pharmaceuticals. C.P. received personal compensation from Novartis and has acted as an investigator in clinical trials for Santhera Pharmaceuticals, GenSight Biologics, and Iveric Bio. X.L. and L.T. are employees of Chiesi Farmaceutici S.p.A.

Received: July 3, 2023
Revised: October 24, 2023
Accepted: January 30, 2024
Published: February 29, 2024

REFERENCES

1. Carelli, V., Carbonelli, M., de Coo, I.F., Kawasaki, A., Klopstock, T., Lagrèze, W.A., La Morgia, C., Newman, N.J., Orssaud, C., Pott, J.W.R., et al. (2017). International consensus statement on the clinical and therapeutic management of Leber hereditary optic neuropathy. J. Neuro Ophthalmol. *37*, 371–381. https://doi.org/10.1097/WNO.0000000000000570.
2. Zeviani, M., and Carelli, V. (2021). Mitochondrial retinopathies. Int. J. Mol. Sci. *23*, 210. https://doi.org/10.3390/ijms23010210.
3. Yu-Wai-Man, P., Griffiths, P.G., Hudson, G., and Chinnery, P.F. (2009). Inherited mitochondrial optic neuropathies. J. Med. Genet. *46*, 145–158. https://doi.org/10.1136/jmg.2007.054270.
4. Gueven, N. (2014). Optic neurodegeneration: Time to act. Biol. Med. *01*. https://doi.org/10.4172/0974-8369.S1-001.
5. Riordan-Eva, P., Sanders, M.D., Govan, G.G., Sweeney, M.G., Da Costa, J., and Harding, A.E. (1995). The clinical features of Leber's hereditary optic neuropathy defined by the presence of a pathogenic mitochondrial DNA mutation. Brain *118*, 319–337. https://doi.org/10.1093/brain/118.2.319.
6. Jiang, P., Liang, M., Zhang, J., Gao, Y., He, Z., Yu, H., Zhao, F., Ji, Y., Liu, X., Zhang, M., et al. (2015). Prevalence of Mitochondrial ND4 Mutations in 1281 Han Chinese Subjects With Leber's Hereditary Optic Neuropathy. Invest. Ophthalmol. Vis. Sci. *56*, 4778–4788. https://doi.org/10.1167/iovs.14-16158.
7. Yen, M.-Y., Wang, A.-G., and Wei, Y.-H. (2006). Leber's hereditary optic neuropathy: a multifactorial disease. Prog. Retin. Eye Res. *25*, 381–396. https://doi.org/10.1016/j.preteyeres.2006.05.002.
8. Chalmers, R.M., and Harding, A.E. (1996). A case-control study of Leber's hereditary optic neuropathy. Brain *119*, 1481–1486.
9. Jiang, P., Liang, M., Zhang, C., Zhao, X., He, Q., Cui, L., Liu, X., Sun, Y.-H., Fu, Q., Ji, Y., et al. (2016). Biochemical evidence for a mitochondrial genetic modifier in the phenotypic manifestation of Leber's hereditary optic neuropathy-associated mitochondrial DNA mutation. Hum. Mol. Genet. *25*, 3613–3625. https://doi.org/10.1093/hmg/ddw199.
10. Cheng, H.-C., Chi, S.-C., Liang, C.-Y., Yu, J.-Y., and Wang, A.-G. (2022). Candidate Modifier Genes for the Penetrance of Leber's Hereditary Optic Neuropathy. Int. J. Mol. Sci. *23*, 11891. https://doi.org/10.3390/ijms231911891.

CellPress
OPEN ACCESS

11. Liang, M., Jiang, P., Li, F., Zhang, J., Ji, Y., He, Y., Xu, M., Zhu, J., Meng, X., Zhao, F., et al. (2014). Frequency and spectrum of mitochondrial ND6 mutations in 1218 Han Chinese subjects with Leber's hereditary optic neuropathy. Invest. Ophthalmol. Vis. Sci. *55*, 1321–1331. https://doi.org/10.1167/iovs.13-13011.

12. Newman, N.J., Yu-Wai-Man, P., Biousse, V., and Carelli, V. (2023). Understanding the molecular basis and pathogenesis of hereditary optic neuropathies: towards improved diagnosis and management. Lancet Neurol. *22*, 172–188. https://doi.org/10.1016/S1474-4422(22)00174-0.

13. Stenton, S.L., Sheremet, N.L., Catarino, C.B., Andreeva, N.A., Assouline, Z., Barboni, P., Barel, O., Berutti, R., Bychkov, I., Caporali, L., et al. (2021). Impaired complex I repair causes recessive Leber's hereditary optic neuropathy. J. Clin. Invest. *131*, e138267. https://doi.org/10.1172/JCI138267.

14. Gerber, S., Ding, M.G., Gérard, X., Zwicker, K., Zanlonghi, X., Rio, M., Serre, V., Hanein, S., Munnich, A., Rotig, A., et al. (2017). Compound heterozygosity for severe and hypomorphic NDUFS2 mutations cause non-syndromic LHON-like optic neuropathy. J. Med. Genet. *54*, 346–356. https://doi.org/10.1136/jmedgenet-2016-104212.

15. Kieninger, S., Xiao, T., Weisschuh, N., Kohl, S., Rüther, K., Kroisel, P.M., Brockmann, T., Knappe, S., Kellner, U., Lagrèze, W., et al. (2022). DNAJC30 disease-causing gene variants in a large Central European cohort of patients with suspected Leber's hereditary optic neuropathy and optic atrophy. J. Med. Genet. *59*, 1027–1034. https://doi.org/10.1136/jmedgenet-2021-108235.

16. Fiorini, C., Degiorgi, A., Cascavilla, M.L., Tropeano, C.V., La Morgia, C., Battista, M., Ormanbekova, D., Palombo, F., Carbonelli, M., Bandello, F., et al. (2023). Recessive MECR pathogenic variants cause an LHON-like optic neuropathy. J. Med. Genet. *61*, 93–101. https://doi.org/10.1136/jmg-2023-109340.

17. Amore, G., Romagnoli, M., Carbonelli, M., Barboni, P., Carelli, V., and La Morgia, C. (2021). Therapeutic Options in Hereditary Optic Neuropathies. Drugs *81*, 57–86. https://doi.org/10.1007/s40265-020-01428-3.

18. Chen, B.S., Yu-Wai-Man, P., and Newman, N.J. (2022). Developments in the Treatment of Leber Hereditary Optic Neuropathy. Curr. Neurol. Neurosci. Rep. *22*, 881–892. https://doi.org/10.1007/s11910-022-01246-y.

19. Gueven, N., and Faldu, D. (2013). Idebenone treatment in Leber's hereditary optic neuropathy: rationale and efficacy. Expert Opin. Orphan Drugs *1*, 331–339. https://doi.org/10.1517/21678707.2013.772894.

20. Haefeli, R.H., Erb, M., Gemperli, A.C., Robay, D., Courdier Fruh, I., Anklin, C., Dallmann, R., and Gueven, N. (2011). NQO1-dependent redox cycling of idebenone: effects on cellular redox potential and energy levels. PLoS One *6*, e17963. https://doi.org/10.1371/journal.pone.0017963.

21. Gueven, N., Ravishankar, P., Eri, R., and Rybalka, E. (2021). Idebenone: When an antioxidant is not an antioxidant. Redox Biol. *38*, 101812. https://doi.org/10.1016/j.redox.2020.101812.

22. Danese, A., Patergnani, S., Maresca, A., Peron, C., Raimondi, A., Caporali, L., Marchi, S., La Morgia, C., Del Dotto, V., Zanna, C., et al. (2022). Pathological mitophagy disrupts mitochondrial homeostasis in Leber's hereditary optic neuropathy. Cell Rep. *40*, 111124. https://doi.org/10.1016/j.celrep.2022.111124.

23. Klopstock, T., Yu-Wai-Man, P., Dimitriadis, K., Rouleau, J., Heck, S., Bailie, M., Atawan, A., Chattopadhyay, S., Schubert, M., Garip, A., et al. (2011). A randomized placebo-controlled trial of idebenone in Leber's hereditary optic neuropathy. Brain *134*, 2677–2686. https://doi.org/10.1093/brain/awr170.

24. Catarino, C.B., von Livonius, B., Priglinger, C., Banik, R., Matloob, S., Tamhankar, M.A., Castillo, L., Friedburg, C., Halfpenny, C.A., Lincoln, J.A., et al. (2020). Real-world clinical experience with idebenone in the treatment of Leber hereditary optic neuropathy. J. Neuro Ophthalmol. *40*, 558–565. https://doi.org/10.1097/WNO.0000000000001023.

25. Newman, N.J., Carelli, V., Taiel, M., and Yu-Wai-Man, P. (2020). Visual outcomes in Leber hereditary optic neuropathy patients with the m.11778GA (MTND4) mitochondrial DNA mutation. J. Neuro Ophthalmol. *40*, 547–557. https://doi.org/10.1097/WNO.0000000000001045.

26. Yu-Wai-Man, P., Newman, N.J., Carelli, V., La Morgia, C., Biousse, V., Bandello, F.M., Clermont, C.V., Campillo, L.C., Leruez, S., Moster, M.L., et al. (2022). Natural history of patients with Leber hereditary optic neuropathy-results from the REALITY study. Eye (Lond) *36*, 818–826. https://doi.org/10.1038/s41433-021-01535-9.

27. Pemp, B., Kircher, K., and Reitner, A. (2019). Visual function in chronic Leber's hereditary optic neuropathy during idebenone treatment initiated 5 to 50 years after onset. Graefes Arch. Clin. Exp. Ophthalmol. *257*, 2751–2757. https://doi.org/10.1007/s00417-019-04444-6.

28. Pemp, B., Mitsch, C., Kircher, K., and Reitner, A. (2021). Changes in visual function and correlations with inner retinal structure in acute and chronic Leber's hereditary optic neuropathy patients after treatment with idebenone. J. Clin. Med. *10*, 151. https://doi.org/10.3390/jcm10010151.

29. Newman, N.J. (2005). Hereditary optic neuropathies: from the mitochondria to the optic nerve. Am. J. Ophthalmol. *140*, 517–523. https://doi.org/10.1016/j.ajo.2005.03.017.

30. Ishikawa, H., Masuda, Y., Ishikawa, H., Shikisima, K., Goseki, T., Kezuka, T., Terao, M., Miyazaki, A., Matsumoto, K., Nishikawa, H., et al. (2021). Characteristics of Japanese patients with Leber's hereditary optic neuropathy and idebenone trial: a prospective, interventional, non-comparative study. Jpn. J. Ophthalmol. *65*, 133–142. https://doi.org/10.1007/s10384-020-00789-2.

31. van Everdingen, J.A.M., Pott, J.W.R., Bauer, N.J.C., Krijnen, A.M., Lushchyk, T., and Wubbels, R.J. (2022). Clinical outcomes of treatment with idebenone in Leber's hereditary optic neuropathy in the Netherlands: A national cohort study. Acta Ophthalmol. *100*, 700–706. https://doi.org/10.1111/aos.15153.

32. Mascialino, B., Leinonen, M., and Meier, T. (2012). Meta-analysis of the prevalence of Leber hereditary optic neuropathy mtDNA mutations in Europe. Eur. J. Ophthalmol. *22*, 461–465. https://doi.org/10.5301/ejo.5000055.

33. Guo, D.-Y., Wang, X.-W., Hong, N., and Gu, Y.-S. (2016). A meta-analysis of the association between different genotypes (G11778A, T14484C and G3460A) of Leber hereditary optic neuropathy and visual prognosis. Int. J. Ophthalmol. *9*, 1493–1498. https://doi.org/10.18240/ijo.2016.10.21.

34. Spruijt, L., Kolbach, D.N., de Coo, R.F., Plomp, A.S., Bauer, N.J., Smeets, H.J., and de Die-Smulders, C.E.M. (2006). Influence of mutation type on clinical expression of Leber hereditary optic neuropathy. Am. J. Ophthalmol. *141*, 676–682. https://doi.org/10.1016/j.ajo.2005.11.007.

35. Barboni, P., Savini, G., Valentino, M.L., Montagna, P., Cortelli, P., De Negri, A.M., Sadun, F., Bianchi, S., Longanesi, L., Zanini, M., et al. (2005). Retinal nerve fiber layer evaluation by optical coherence tomography in Leber's Hereditary Optic Neuropathy. Ophthalmology *112*, 120–126. https://doi.org/10.1016/j.ophtha.2004.06.034.

36. Johns, D.R., Smith, K.H., and Miller, N.R. (1992). Leber's hereditary optic neuropathy. Clinical manifestations of the 3460 mutation. Arch. Ophthalmol. *110*, 1577–1581. https://doi.org/10.1001/archopht.1992.01080230077025.

37. Mashima, Y., Kigasawa, K., Shinoda, K., Wakakura, M., and Oguchi, Y. (2017). Visual prognosis better in eyes with less severe reduction of visual acuity one year after onset of Leber hereditary optic neuropathy caused by the 11,778 mutation. BMC Ophthalmol. *17*, 192. https://doi.org/10.1186/s12886-017-0583-3.

38. Varricchio, C., Beirne, K., Heard, C., Newland, B., Rozanowska, M., Brancale, A., and Votruba, M. (2020). The ying and yang of idebenone: Not too little, not too much - cell death in NQO1 deficient cells and the mouse retina. Free Radic. Biol. Med. *152*, 551–560. https://doi.org/10.1016/j.freeradbiomed.2019.11.030.

39. Jaber, S.M., Ge, S.X., Milstein, J.L., VanRyzin, J.W., Waddell, J., and Polster, B.M. (2020). Idebenone has distinct effects on mitochondrial respiration in cortical astrocytes compared to cortical neurons due to differential

NQO1 activity. J. Neurosci. *40*, 4609–4619. https://doi.org/10.1523/JNEUROSCI.1632-17.2020.

40. Aleo, S.J., Del Dotto, V., Romagnoli, M., Fiorini, C., Capirossi, G., Peron, C., Maresca, A., Caporali, L., Capristo, M., Tropeano, C.V., et al. (2024). Genetic variants affecting NQO1 protein levels impact the efficacy of idebenone treatment in Leber hereditary optic neuropathy. Cell Rep. Med. *5*, 101383. https://doi.org/10.1016/j.xcrm.2023.101383.

41. Pan, S.-S., Han, Y., Farabaugh, P., and Xia, H. (2002). Implication of alternative splicing for expression of a variant NAD(P)H:quinone oxidoreductase-1 with a single nucleotide polymorphism at 465CT. Pharmacogenetics *12*, 479–488. https://doi.org/10.1097/00008571-200208000-00009.

42. Siegel, D., Anwar, A., Winski, S.L., Kepa, J.K., Zolman, K.L., and Ross, D. (2001). Rapid polyubiquitination and proteasomal degradation of a mutant form of NAD(P)H:quinone oxidoreductase 1. Mol. Pharmacol. *59*, 263–268. https://doi.org/10.1124/mol.59.2.263.

43. Lienhart, W.-D., Gudipati, V., Uhl, M.K., Binter, A., Pulido, S.A., Saf, R., Zangger, K., Gruber, K., and Macheroux, P. (2014). Collapse of the native structure caused by a single amino acid exchange in human NAD(P)H:quinone oxidoreductase(1.). FEBS J. *281*, 4691–4704. https://doi.org/10.1111/febs.12975.

44. Majander, A., Bowman, R., Poulton, J., Antcliff, R.J., Reddy, M.A., Michaelides, M., Webster, A.R., Chinnery, P.F., Votruba, M., Moore, A.T., and Yu-Wai-Man, P. (2017). Childhood-onset Leber hereditary optic neuropathy. Br. J. Ophthalmol. *101*, 1505–1509. https://doi.org/10.1136/bjophthalmol-2016-310072.

45. Pezzi, P.P., De Negri, A.M., Sadun, F., Carelli, V., and Leuzzi, V. (1998). Childhood Leber's hereditary optic neuropathy (ND1/3460) with visual recovery. Pediatr. Neurol. *19*, 308–312. https://doi.org/10.1016/s0887-8994(98)00060-5.

46. Klopstock, T., Llòria, X., Silva, M., Catarino, C.B., Lob, F., Livonius, B. von, and Rudolph, G. (2019). Poster presentation PO53. Responder analysis of chronic Leber's hereditary optic neuropathy (LHON) patients to idebenone in a placebo controlled, randomised clinical trial. Neuro Ophthalmol. *43*, 1–221. https://doi.org/10.1080/01658107.2019.1608780.

47. Barboni, P., La Morgia, C., Cascavilla, M.L., Hong, E.H., Battista, M., Majander, A., Caporali, L., Starace, V., Amore, G., Renzo, A.D., et al. (2023). Childhood-Onset Leber Hereditary Optic Neuropathy-Clinical and Prognostic Insights. Am. J. Ophthalmol. *249*, 99–107. https://doi.org/10.1016/j.ajo.2022.12.014.

48. Fantini, M., Asanad, S., Karanjia, R., and Sadun, A. (2019). Hormone replacement therapy in Leber's hereditary optic neuropathy: Accelerated visual recovery in vivo. J. Curr. Ophthalmol. *31*, 102–105. https://doi.org/10.1016/j.joco.2018.10.003.

49. Giordano, C., Montopoli, M., Perli, E., Orlandi, M., Fantin, M., Ross-Cisneros, F.N., Caparrotta, L., Martinuzzi, A., Ragazzi, E., Ghelli, A., et al. (2011). Oestrogens ameliorate mitochondrial dysfunction in Leber's hereditary optic neuropathy. Brain *134*, 220–234. https://doi.org/10.1093/brain/awq276.

50. Pisano, A., Preziuso, C., Iommarini, L., Perli, E., Grazioli, P., Campese, A.F., Maresca, A., Montopoli, M., Masuelli, L., Sadun, A.A., et al. (2015). Targeting estrogen receptor β as preventive therapeutic strategy for Leber's hereditary optic neuropathy. Hum. Mol. Genet. *24*, 6921–6931. https://doi.org/10.1093/hmg/ddv396.

51. Newman, N.J., Yu-Wai-Man, P., Carelli, V., Biousse, V., Moster, M.L., Vignal-Clermont, C., Sergott, R.C., Klopstock, T., Sadun, A.A., Girmens, J.-F., et al. (2021). Intravitreal gene therapy vs. natural history in patients with Leber hereditary optic neuropathy carrying the m.11778GA ND4 mutation: Systematic review and indirect comparison. Front. Neurol. *12*, 662838. https://doi.org/10.3389/fneur.2021.662838.

52. Kaiser, P.K. (2009). Prospective Evaluation of Visual Acuity Assessment: A Comparison of Snellen versus ETDRS Charts in Clinical Practice (An AOS Thesis) (Trans Am Ophthalmol Soc), pp. 311–324.

53. Metz, G., Coppard, N., Petraki, D., Meier, T., Klopstock, T., and Sahel, J. (2014). A case report survey (CRS) on the natural history of visual acuity in patients with Leber's hereditary optic neuropathy (LHON). Acta Ophthalmol. *92*. https://doi.org/10.1111/j.1755-3768.2014.T110.x.

54. Silva, M., Lloria, X., Catarino, C., Klopstock, T., and Sahel, J.-A. (2018). Natural History Data (NHD) In A Cohort Of 383 Patients with Leber's Hereditary Optic Neuropathy (LHON). Results From An International Retrospective Case Record Survey (CRS). Invest. Ophthalmol. Vis. Sci. *59*, 3362.

55. European Medicines Agency (2001). ICH E10 Choice of Control Group and Related Issues in Clinical Trials. https://www.ema.europa.eu/en/documents/scientific-guideline/ich-e-10-choice-control-group-clinical-trials-step-5_en.pdf.

56. Silva, M., Llòria, X., Catarino, C.B., and Klopstock, T. (2018). Natural history findings from a large cohort of patients with Leber's hereditary optic neuropathy (LHON): New insights into the natural disease-course. Acta Ophthalmol. *96*, 117. https://doi.org/10.1111/aos.13972_438.

STAR★METHODS

KEY RESOURCES TABLE

REAGENT or RESOURCE	SOURCE	IDENTIFIER
Chemicals, peptides, and recombinant proteins		
Idebenone	Chiesi Farmaceutici S.p.A	Raxone®
Deposited data		
Patient data	This manuscript	ClinicalTrials.gov NCT02774005
Software and algorithms		
SAS	SAS Institute	Version 9.4
nQuery Advisor	Statsols	Version 8.3

RESOURCE AVAILABILITY

Lead contact

Further information and requests for resources should be directed to and will be fulfilled by the lead contact, Thomas Klopstock (thomas.klopstock@med.uni-muenchen.de).

Materials availability

This study did not generate new unique reagents.

Data and code availability

- The individual patient data reported in this study cannot be deposited in a public repository because these data are confidential medical records. To request access, please contact Thomas Klopstock (thomas.klopstock@med.uni-muenchen.de) for de-identified summary data.
- This paper does not report original code.
- Any additional information required to reanalyze the data reported in this paper is available from the lead contact upon request.

EXPERIMENTAL MODEL AND SUBJECT DETAILS

An Institutional Review Board or Independent Ethics Committee reviewed and approved the study protocol and any amendments prior to their implementation, the informed consent forms and their updates, and any written materials given to patients at each site. All patients provided informed written consent to participate before any study activity was initiated. All procedures were followed in accordance with the ethical principles of the Declaration of Helsinki, and in compliance with the approved protocol, Good Clinical Practice guidelines, and applicable regulatory requirements.

Patients with LHON were enrolled in this study. Information on the sample size and demographics of the idebenone-treated population (LEROS) is provided in Table 1, Table S4 and Figure S1. Information on the sample size and demographics of the Natural History (NH) population is provided in Table 1, Table S1 and Figure S2.

METHOD DETAILS

Study design and objectives

LEROS was an international, multicenter, Phase IV, open-label interventional study to assess the efficacy and safety of idebenone in LHON relative to an idebenone-naïve, external NH cohort. In LEROS, VA assessments were performed at follow-up visits 1, 3, 6, 12, 18 and 24 months after the baseline visit.

Sample size determination

LEROS sample size was based on the primary endpoint, clinically relevant benefit (CRB) from baseline at 12 months in the eyes of patients with LHON in the subacute/dynamic phase. The initial sample size calculation for LEROS assumed an expected 24% responder rate in the external NH control group and 40% in the idebenone-treated population. A total of 177 eyes per group were needed to demonstrate a CRB in favor of idebenone, with 90% power and 5% alpha 2-sided binomial test with normal approximation. Once the enrollment of CRS-2 was completed, a pre-planned check of the estimate of responder rate in the control group was performed, using combined data from 175 eyes in the two NH studies. The updated estimated responder rate was a maximum of

22%. A sample size re-calculation using the updated control group estimate and maintaining all other previous assumptions, determined that 137 eyes per group were enough to demonstrate a CRB in favor of idebenone, with an assumed ratio of idebenone-treated and NH patients of 1:1. In order to account for a drop-out rate of 30%, at least 80 patients (equal to 160 eyes) were enrolled to the LEROS study. The sample size calculation was performed using nQuery Advisor version 8.3.

Eligibility criteria and study populations

Treatment-naïve patients $\geq$12 years of age with LHON-related impaired vision and symptom onset $\leq$5 years prior to baseline were enrolled. Patients who had previously provided NH data to the CRS-1[53,54] (these studies were registered at ClinicalTrials.gov: NCT01892943) and −2 (CRS-2, ClinicalTrials.gov: NCT02796274), used idebenone, were pregnant or breastfeeding, had participated in another clinical trial of any investigational drug $\leq$3 months prior to the baseline visit, or had a known history of abnormal liver function tests were not eligible for enrollment. Patients with LHON caused by the m.11778G>A mutation who had undergone gene therapy for LHON over 3 months before participating in LEROS were not excluded from participation. Nevertheless, no use of such therapy was recorded in any of the patients in this study. Enrolled patients received 900 mg/day idebenone (2 × 150 mg; orally three times/day) for up to 24 months from the baseline visit.

The ITT population included all enrolled patients undergoing at least one post-baseline VA assessment. The modified-ITT (mITT) population was used to evaluate efficacy endpoints relative to the NH cohort and only included patients carrying one of the three most common causative mtDNA mutations (m.11778G>A, m.3460G>A, and m.14484T>C).

Natural history comparator population

Background and rationale

The generation of a combined dataset of natural history data from two case record surveys was performed as a post-authorization measure in response to a request from the European Medicines Agency (EMA) to provide controlled efficacy data to support the marketing authorization of idebenone under exceptional circumstances. The EMA acknowledged that a placebo-controlled study in newly diagnosed patients is not feasible considering the rarity of LHON and the fact that idebenone is already available on the market. The regulators agreed that the comparison of treatment outcomes with natural history data from a prospectively defined, external, untreated “Natural History” control group dataset represents a feasible option in a rare disease with high unmet medical need such as LHON.

Data for the external control group were obtained as secondary data from two retrospective case record surveys, CRS-1 (NCT01892943)[53,54] and CRS-2 (NCT02796274), the latter being designed specifically to contribute to the external control group for LEROS. The proposed methodology for generating the external control dataset was reviewed by the EMA as part of the regulatory pathway toward marketing authorization and no issues were raised.

Limitations around the use of external historical control data

The use of external controls is associated with potential limitations that have been well described.[55] For example, untreated historical control groups tend to have worse outcomes than apparently similarly chosen control groups in randomized studies, possibly reflecting a selection bias. However, strategies to increase the assurance of comparability between patient groups and to reduce bias, such as pre-specification of patient selection and matching criteria, can be implemented to improve the interpretability of externally controlled study outcomes.[55] To optimize the comparability of the LEROS data with the combined case record survey control data, the control dataset was prospectively established, and a matching algorithm was applied to select baseline datapoints only after LEROS was finished and baseline characteristics were known (see section Matching algorithm). In this way, bias in the selection of control data for comparison with the outcomes of the open label study was minimized.

Design

The external NH control set consisted of combined data from two international, multicenter historical case record surveys; CRS-1[53,54,56] (ClinicalTrials.gov: NCT01892943) and CRS-2 (ClinicalTrials.gov: NCT02796274). CRS-1 was a retrospective medical record survey conducted at ten sites across Europe and one site in the USA from May 2013 to February 2014. Participating clinical centers were asked to provide historical case record data from all LHON patients with a molecular diagnosis on file without pre-selection. CRS-2 was a retrospective medical record survey conducted at 20 sites across seven countries in Europe from May 2016 to March 2018. It included data from existing medical records of patients who were $\geq$12 years old, had one of the three most common mitochondrial DNA (mtDNA) mutations, experienced onset of symptoms after 1999 with the month of onset for both eyes known, and at least two VA assessments with defined visit dates available within 5 years of onset and prior to idebenone use. Patients who had participated in an interventional clinical trial after the onset of symptoms, or who had any other cause of visual impairment or any active ocular disorder during the data collection period, were excluded.

Matching algorithm

In LHON, the time since onset of symptoms is a major factor influencing VA outcomes. A matching algorithm was applied to ensure that the time from symptom onset to baseline assessment was comparable between idebenone-treated eyes and eyes from the NH cohort. The proposed methodology for generating the external control dataset was reviewed by the EMA as part of the regulatory pathway toward marketing authorization and no issues were raised. Different matching algorithms were used for eyes in the subacute/dynamic and chronic phases.

For subacute/dynamic eyes, the average time from onset of symptoms in the second eye to the baseline visit was calculated ($onset_L$). Since there was no treatment in the NH control set, in principle any VA observation at any timepoint after the onset of symptoms could be used as a baseline VA for a given eye. Eyes with a possible baseline visit within 1 year from symptom onset were selected following identification of visit pairs in which a follow-up visit occurred within a window of ±3 months of each assessment timepoint (e.g., 12 ± 3 months and 24 ± 3 months) (Figure S6). Of these visit pairs, that which was closest to $onset_L$ was selected for the matched NH control group.

In chronic eyes, visit pairs were determined in the NH cohort, with the first VA assessment of each pair qualifying for a potential baseline visit. Visit pairs were categorized into bins of >1 to 2, >2 to 3, >3 to 4, and >4 to 5 years according to the time from symptom onset at each possible baseline timepoint. One eye could have a visit pair in each bin. In case several visit pairs fell in the same bin, the baseline value closest to the midpoint of the bin was selected.

QUANTIFICATION AND STATISTICAL ANALYSIS

Definition of outcome measures

In LEROS, VA was determined using Early Treatment Diabetic Retinopathy Study (ETDRS) charts and recorded in logarithm of the minimum angle of resolution (logMAR). The conversion of VA measurements in the NH group to logMAR values is described in the subsection "Conversion of VA measurements to logMAR values" below and in Table S3. Off-chart subcategories (i.e., "counting fingers", "hand motion", "light perception", "no light perception") or changes within these subcategories were not analyzed separately in LEROS, but were all assigned a value of 1.8 logMAR, consistent with previous idebenone studies.

Treatment efficacy was assessed using four VA response measures: clinically relevant benefit (CRB), clinically relevant recovery (CRR), clinically relevant stabilization (CRS), and clinically relevant worsening (CRW) (Figure S7). CRB was a composite measure considered as a CRR and/or a CRS. CRR was defined as improvement from an off-chart VA to reading at least 5 letters on-chart (≤1.6 logMAR), or improvement of at least 10 additional letters (−0.2 logMAR) for those already on-chart. CRS was defined as maintenance of VA <1.0 logMAR from baseline to the visit timepoint. CRW was defined as a worsening from on-chart to off-chart, or a loss of at least 10 letters (+0.2 logMAR) on-chart. The primary endpoint was the rate of CRB from baseline at 12 months in idebenone-treated, subacute/dynamic eyes versus matched eyes of the NH cohort.

Conversion of VA measurements to logMAR values

In the NH control group, VA was recorded using ETDRS charts, Snellen charts, or decimal scores. Snellen values were converted to decimal scores, and in turn to logMAR values using the formula -log(decimal acuity).

Statistical methods

The primary endpoint and all other CRB, CRR, CRS, and CRW outcomes were analyzed using a logistic regression model, including treatment, gender and mutation as fixed factors. Change in VA from baseline was analyzed using analysis of covariance (ANCOVA) with VA at baseline as a covariate and fixed factors of treatment, gender, and mutation. For the analyses stratified by age group, the same model was used, with the addition of age group and treatment-age group interaction. Time to CRR in the ITT populations was evaluated using Kaplan-Meier estimates and curves. Except for the primary endpoint, to which the study was powered, all p values were calculated as exploratory. Data analyses were performed using SAS version 9.4 (SAS Institute).

ADDITIONAL RESOURCES

This study was registered on ClinicalTrials.gov (NCT02774005).

Cell Reports Medicine, Volume *5*

Supplemental information

Therapeutic benefit of idebenone in patients with Leber hereditary optic neuropathy: The LEROS nonrandomized controlled trial

Patrick Yu-Wai-Man, Valerio Carelli, Nancy J. Newman, Magda Joana Silva, Aki Linden, Gregory Van Stavern, Jacek P. Szaflik, Rudrani Banik, Wojciech Lubiński, Berthold Pemp, Yaping Joyce Liao, Prem S. Subramanian, Marta Misiuk-Hojło, Steven Newman, Lorena Castillo, Jarosław Kocięcki, Marc H. Levin, Francisco Jose Muñoz-Negrete, Ali Yagan, Sylvia Cherninkova, David Katz, Audrey Meunier, Marcela Votruba, Magdalena Korwin, Jacek Dziedziak, Neringa Jurkutė, Joshua P. Harvey, Chiara La Morgia, Claudia Priglinger, Xavier Llòria, Livia Tomasso, Thomas Klopstock, and LEROS Study Group

Supplemental Information

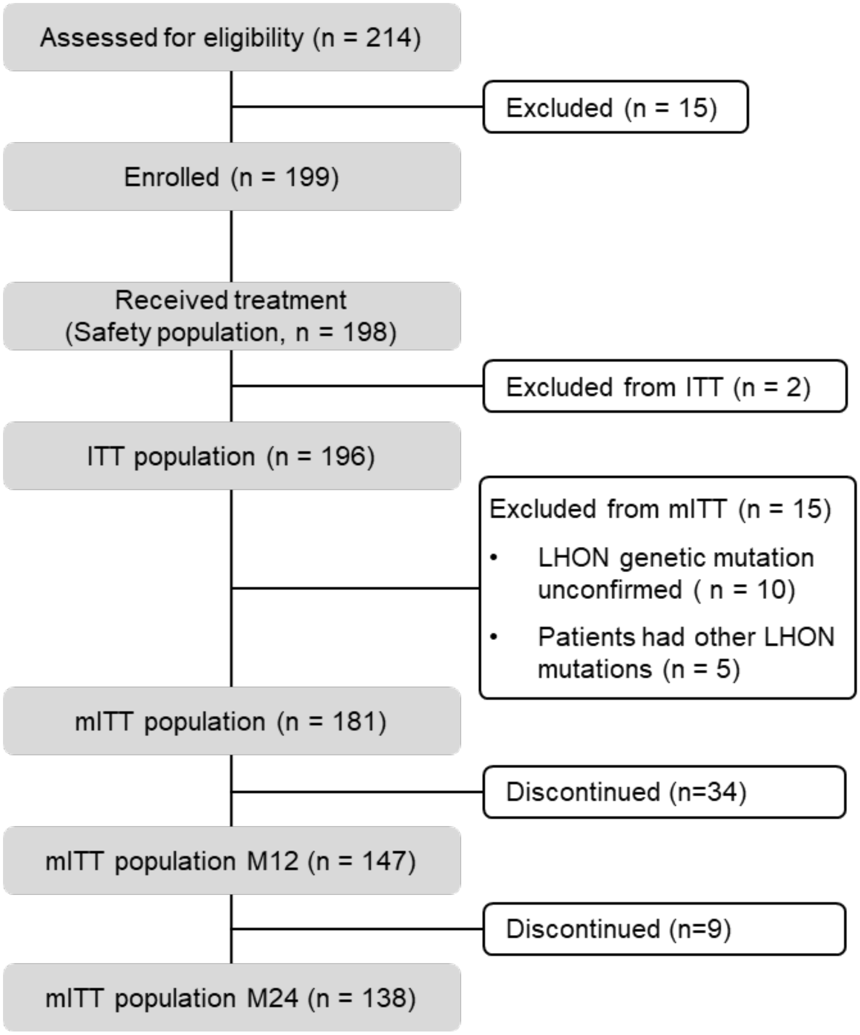

Figure S 1. Disposition of idebenone treated patients, related to Results and STAR Methods
ITT: Intention-To-Treat; LHON: Leber hereditary optic neuropathy; mITT: modified ITT; VA: visual acuity.

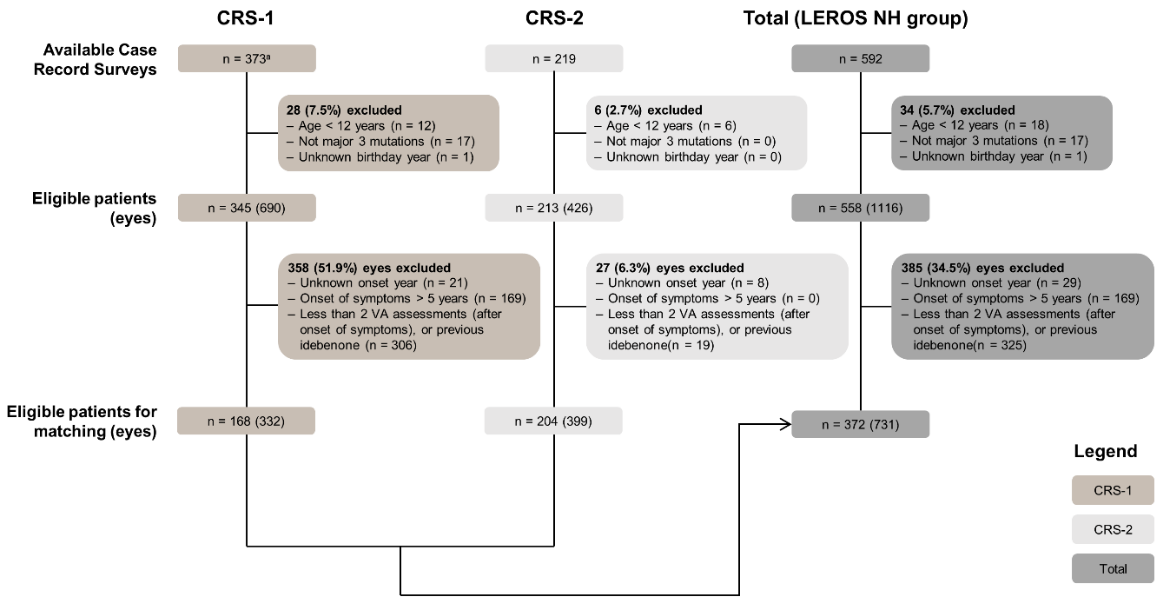

Figure S 2. Disposition of patients in the Natural History control group, related to STAR Methods

[a]Of 383 available record surveys, 10 were of patients included in CRS-2 and were removed from CRS-1. CRS: Case Record Survey; NH: Natural History.

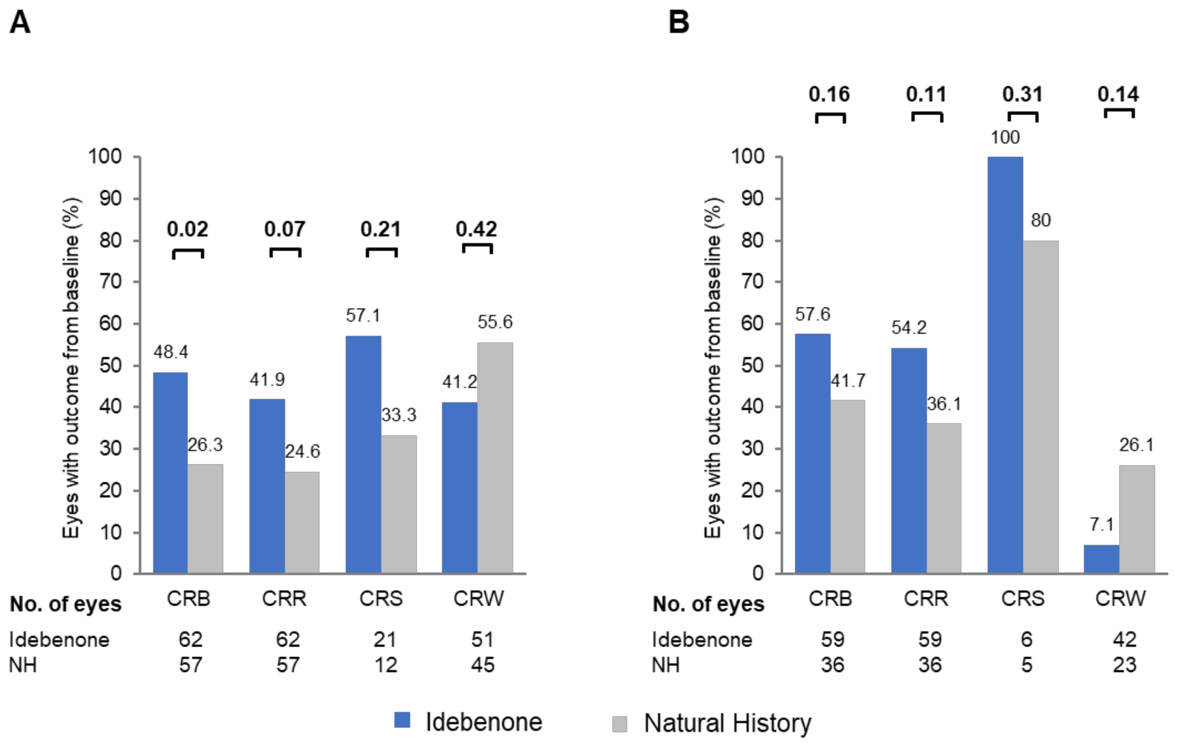

Figure S 3. Responder outcomes at 24 months in (A) subacute eyes (< 6 months after onset at baseline) and (B) dynamic eyes (6 – 12 months after onset at baseline) (mITT vs matched NH). Related to Figure 1
CRB: clinically relevant benefit; CRS: clinically relevant stabilization; CRR: clinically relevant recovery; CRW: clinically relevant worsening; mITT: modified Intention-To-Treat cohort; NH: Natural History cohort.

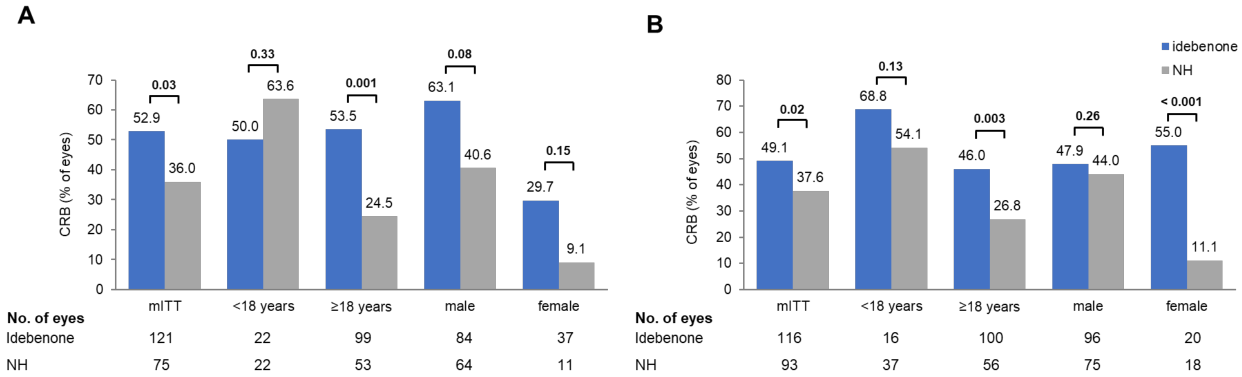

Figure S 4. CRB from baseline by age group at first symptom onset and by gender in (A) subacute/dynamic and (B) chronic eyes at 24 months (mITT vs matched NH). Related to Figure 1
Idebenone had a significant therapeutic benefit in adult eyes (≥18 years) at 24 months (subacute/dynamic [53.5% vs 24.5%, p=0.001]; chronic [46.0% vs 26.8%, p=0.003]). In patients <18 years at symptom onset, the rate of CRB was comparably high in both treated and untreated eyes (subacute/dynamic [50.0% vs 63.6%, p=0.33]; chronic [68.8% vs 54.1%, p=0.13]). Subgroup analyses of CRB by ***gender*** demonstrated a therapeutic benefit of idebenone at 24 months in eyes of female patients in the chronic phase. In the subacute/dynamic phase there was a non-significant trend toward a positive treatment effect in both ***genders***. In subacute/dynamic eyes, the difference in rates was larger in eyes of male patients, whereas this was reversed in the chronic phase. CRB: clinically relevant benefit; mITT: modified Intention-To-Treat cohort; NH: Natural History cohort.

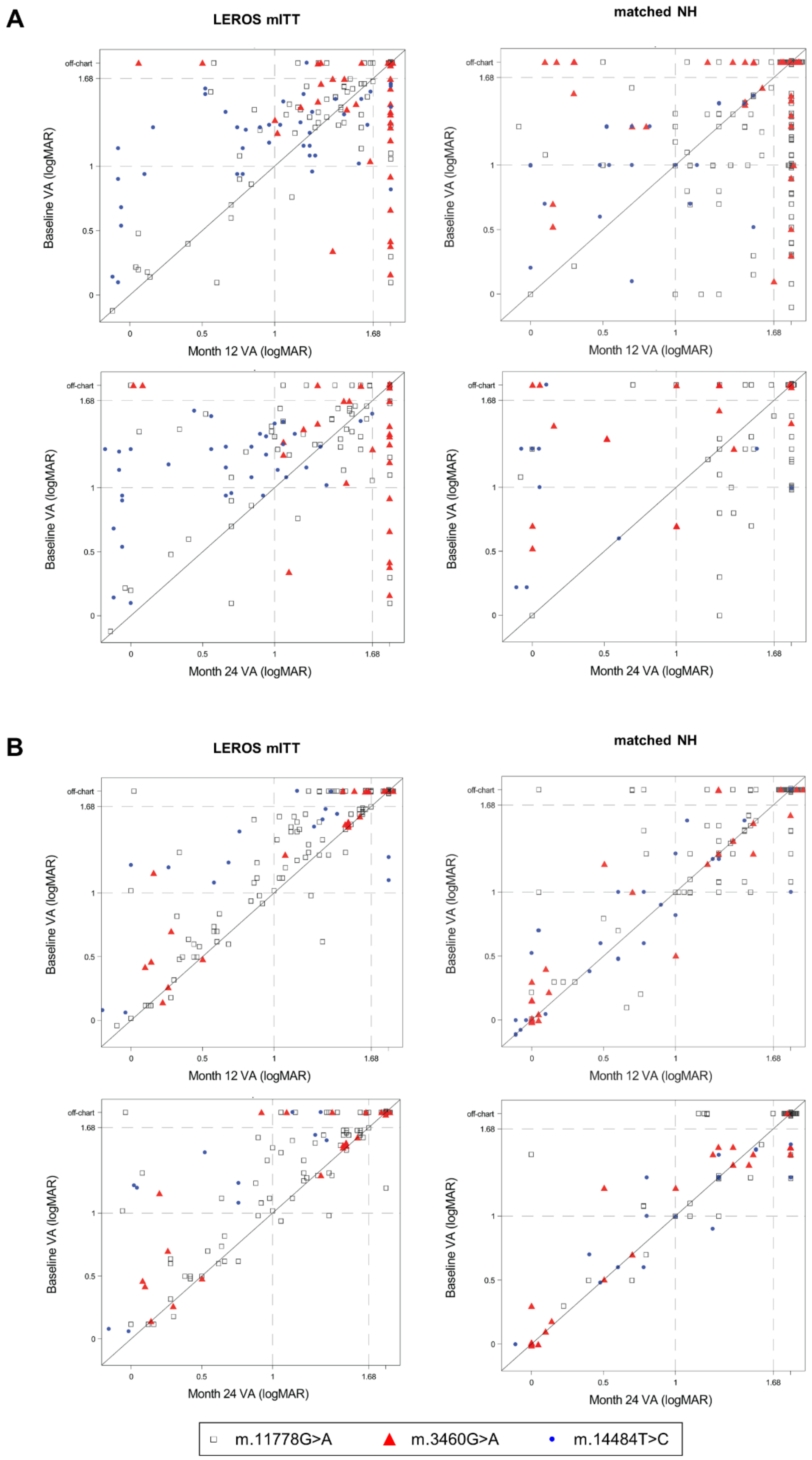

Figure S 5. Visual acuity of individual eyes at baseline versus 12 or 24 months in (A) subacute/dynamic eyes, and (B) chronic eyes (mITT and matched Natural History groups). Related to Figure 4
Off-chart VA was defined as 1.8 logMAR. mITT: modified intention-to-treat; NH: Natural History; VA: visual acuity.

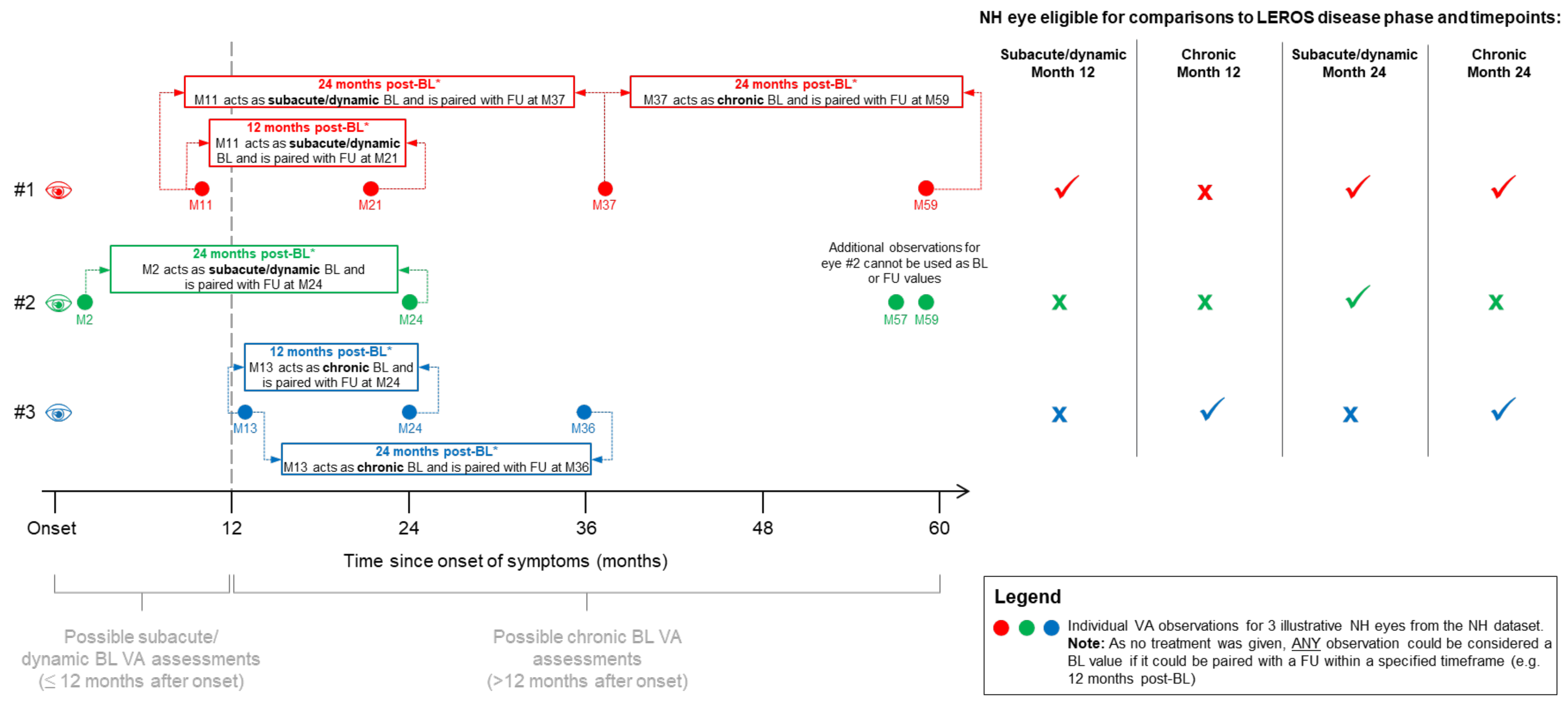

Figure S 6. Illustration of matching subacute/dynamic eyes in the Natural History group to the idebenone-treated LEROS mITT population, related to STAR Methods

* 12 or 24 months post-BL ± 3 months.

BL: baseline; FU: follow-up; NH: Natural History; M: Month; VA: visual acuity.

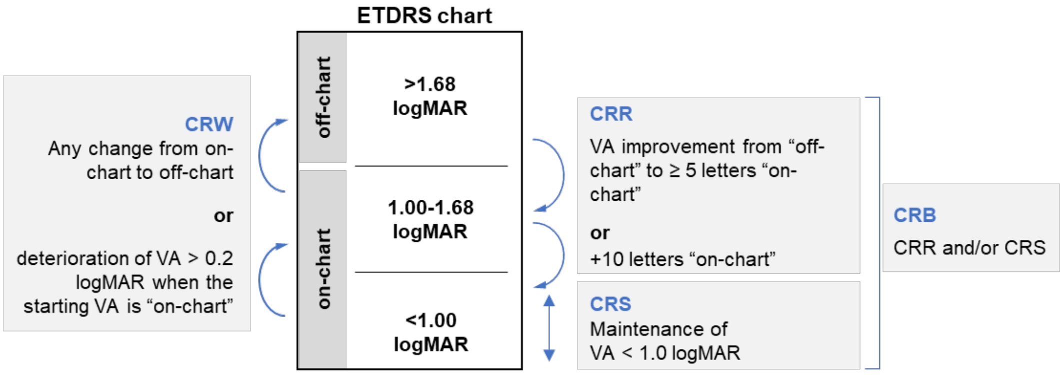

Figure S 7. Definition of outcome measures, related to STAR Methods
CRB: clinically relevant benefit; CRR: clinically relevant recovery; CRS: clinically relevant stabilization; CRW: clinically relevant worsening; ETDRS: Early Treatment Diabetic Retinopathy Study; VA: visual acuity.

Table S 1. Patient characteristics in the different Natural History subsets, related to Table 1

In line with literature reports, around 80% of patients were male with a median age of first symptom onset around 24 – 26 years. [1] The proportions of the three major mutations were also as expected with m.11778G>A found in 73% of patients, followed by 14% carrying the m.3460G>A mutation, and 13% the m.14484T>C mutation in patients eligible for matching.(Yu-Wai-Man et al. 2009) At first symptom onset, 4.6% of patients eligible for matching were <12 years of age and 15.6% were adolescent (12 to <18 years). The majority of patients were adults, with 46.5% in the age group of 18 to <35 years and 33.3% in the age group of 35 years or older. For over 50% of patients eligible for matching at least 5 visits with VA assessments were recorded. Nevertheless, the number of visits was relatively low, leading to a strong reduction of patients with possible baseline-visit pairs for the outcome analyses. Only 36.0% (n=134) of patients eligible for matching had a suitable VA pair at 12 months, and 18.5% (n=69) at 24 months.

**Characteristic**	**(A) Total NH population**	**(B) Patients with ≥ 2 visits prior to idebenone**	**(C) Patients in (B) with known onset year and ≤ 5 years since onset**	**(D) Patients in (C) with one of major 3 mutations**	**(E) Patients eligible for matching (Patients in (D) aged ≥ 12 years and year of birth known)**
**Patients, N**	**592**	**419**	**391**	**383**	**372**
**Gender, n (%)**					
Female	124 (20.9%)	85 (20.3%)	78 (19.9%)	76 (19.8%)	**74 (19.9%)**
Male	462 (78.0%)	333 (79.5%)	312 (79.8%)	306 (79.9%)	**297 (79.8%)**
Missing	6 (1.0%)	1 (0.2%)	1 (0.3%)	1 (0.3%)	**1 (0.3%)**
**Mutation, n (%)**					
m.11778G>A	404 (68.2%)	295 (70.4%)	279 (71.4%)	279 (72.8%)	**273 (73.4%)**
m.3460G>A	95 (16.0%)	59 (14.1%)	55 (14.1%)	55 (14.4%)	**52 (14.0%)**
m.14484T>C	76 (12.8%)	54 (12.9%)	49 (12.5%)	49 (12.8%)	**47 (12.6%)**
Other	17 (2.9%)	11 (2.6%)	8 (2.0%)	-	**-**
**Age (years) at 1st symptom onset (years), N**	581	413	390	382	**372**
Mean (SD)	28 (14.6)	29 (14.6)	29 (14.6)	29 (14.6)	**30 (14.3)**
Median (Q1; Q3)	24 (17; 37)	25 (18; 38)	25 (18; 39)	26 (18; 39)	**26 (18; 39)**
Min – max	4, 78	4, 75	4, 75	4, 75	**4, 75**
**Age group at 1st symptom onset, n (%)**					
<12 years	44 (7.4%)	33 (7.9%)	28 (7.2%)	27 (7.0%)	**17 (4.6%)**
12 ≤ years <18	102 (17.2%)	69 (16.5%)	60 (15.3%)	58 (15.1%)	**58 (15.6%)**
18 ≤ years <35	270 (45.6%)	183 (43.7%)	177 (45.3%)	173 (45.2%)	**173 (46.5%)**
≥ 35 years	165 (27.9%)	128 (30.5%)	125 (32.0%)	124 (32.4%)	**124 (33.3%)**
Missing	11 (1.9%)	6 (1.4%)	1 (0.3%)	1 (0.3%)	**-**
**Number of visits per patient with VA assessment, N**	587	419	391	383	**372**
Mean (SD)	4 (3.5)	5 (3.6)	6 (3.6)	5 (3.6)	**5 (3.6)**
Median (Q1; Q3)	3 (2; 6)	4 (3; 7)	5 (3; 7)	5 (3; 7)	**5 (3; 7)**
Range	1, 31	2, 31	2, 31	2, 31	**2, 31**
1	109 (18.4%)	-	-	-	**-**
2	104 (17.6%)	79 (18.9%)	70 (17.9%)	70 (18.3%)	**68 (18.3%)**
3	94 (15.9%)	81 (19.3%)	74 (18.9%)	74 (19.3%)	**70 (18.8%)**
4	56 (9.5%)	50 (11.9%)	47 (12.0%)	46 (12.0%)	**45 (12.1%)**
5	58 (9.8%)	55 (13.1%)	53 (13.6%)	52 (13.6%)	**50 (13.4%)**
>5	166 (28.0%)	154 (36.8%)	147 (37.6%)	141 (36.8%)	**139 (37.4%)**

SD: standard deviation; Q: quartile; VA: visual acuity.

Table S 2. Follow-up time and time in NH patient population, related to Table 1

	Patients eligible for matching (N=372)
**Follow-up time (months)**	
Mean (SD)	47.9 (72.41)
Median (Q1; Q3)	14.7 (4.6; 67.6)
Range	0.1 – 514.1
**Time since onset of symptoms to first visit (years)**	
Mean (SD)	0.4 (0.91)
Median (Q1; Q2)	0.1 (0.0; 0.4)
Range	0.0 – 11.2
≤1 year since onset, n (%)	337 (90.6)
>1 year since onset, n (%)	35 (9.4)

Q: quartile; SD: standard deviation; VA: visual acuity.

Table S 3. Visual acuity reporting formats in the Natural History dataset eligible for matching, related to STAR Methods

	**Patients eligible for matching (N=372)**
Type of VA recording, F	3999
logMAR, f (%)	509 (12.7)
Off-chart[a], f (%)	804 (20.1)
Decimal, f (%)	579 (14.5)
Snellen, f (%)	2052 (51.3)
Other, f (%)	55 (1.4)

[a] Counting fingers or worse. Off-chart VA was converted to 1.8 logMAR.
F: frequency; f: frequency; VA: visual acuity.

Table S 4. Patient baseline demographics and characteristics in subacute/dynamic and chronic patients (ITT), related to Table 1

Characteristic	Subacute/dynamic patients (N=109)	Chronic patients (N=87)	ITT (N=196)
Mutation, n (%)	109	87	196
m.11778G>A	55 (50.5)	57 (65.5)	112 (57.1)
m.3460G>A	20 (18.3)	15 (17.2)	35 (17.9)
m.14484T>C	24 (22.0)	10 (11.5)	34 (17.3)
Other	2 (1.8)	3 (3.4)	5 (2.6)
No identified mutation	8 (7.3)	2 (2.3)	10 (5.1)
Male, n (%)	78 (71.6)	66 (75.9)	144 (73.5)
Childbearing potential, N	31	21	52
Yes, n (%)	22 (71.0)	13 (61.9)	35 (67.3)
Race, N	109	87	196
Black or African American, n (%)	6 (5.5)	2 (2.3)	8 (4.1)
White, n (%)	36 (33.0)	18 (20.7)	54 (27.6)
Other[a], n (%)	67 (61.5)	67 (76.9)	134 (68.3)
Height (cm), N	104	85	189
Mean (SD)	172.19 (10.47)	170.44 (12.11)	171.41 (11.24)
Min – max	123.50 – 195.01	107.18 – 190.50	107.18 – 195.01
Weight (kg), N	104	85	189
Mean (SD)	74.54 (15.72)	72.74 (15.79)	73.73 (15.73)
Min – max	43.40 – 119.75	34.10 – 115.21	34.10 – 119.75
Age (years), N	109	87	196
Mean (SD)	34.05 (15.06)	34.06 (15.49)	34.06 (15.21)
Min – max	12.56 – 79.23	12.13 – 76.88	12.13 – 79.23
Age at 1st symptom onset (years), N	109	87	196
Mean (SD), years	33.42 (14.98)	31.43 (15.58)	32.53 (15.24)
Min – max	12.05 – 78.17	8.75 – 75.75	8.75 – 78.17
Months since most recent symptom onset, N	109	87	196
Mean (SD)	5.32 (3.04)	30.30 (14.22)	16.41 (15.78)
Min – max	0.07 – 11.86	12.06 – 57.95	0.07 – 57.95
Number of symptomatic eyes per patient, N	109	87	196
One, n (%)	8 (7.3)	3 (3.4)	11 (5.6)
Two, n (%)	101 (92.7)	84 (96.6)	185 (94.4)
Simultaneous onset in both eyes, N	109	87	196
n (%)	41 (37.6)	37 (42.5)	78 (39.8)
Delta onset[b] (months), N	65	47	112
Mean (SD)	4.49 (5.43)	2.57 (2.42)	3.69 (4.51)
Min – max	0.03 – 27.96	0.30 – 11.86	0.03 – 27.96

[a]Other races included 1 (0.9%) Chinese in the subacute/dynamic LHON cohort, and 2 (2.3%) American Indian or Alaska Native, 1 (1.1%) Asian Indian, and 1 (1.1%) Filipino in the chronic LHON cohort. For 66 (60.6%) patients in the subacute/dynamic and 63 (72.4%) in the chronic LHON cohort the race was not specified.[b]Delta onset is the time gap between symptom onset between a patient's two eyes. Eyes that have equal dates onset were not accounted in the calculation of the delta. ITT: intention-to-treat; LHON: Leber hereditary optic neuropathy; max: maximum; min: minimum; SD: standard deviation.

Table S 5. Responder analysis outcomes in subacute/dynamic and chronic eyes (mITT vs matched NH group), related to Figure 1
The sensitivity analysis takes into consideration additional covariates of age at first symptom onset and time since symptom onset, in addition to treatment, mutations, and gender.

	**All (mITT)**							
	**12 months**				**24 months**			
	**ide,** n/N (%)	**NH,** n/N (%)	***p*-Value; OR [95% CI]**	***Sensitivity analysis* *p*-Value; OR [95% CI]**	**ide,** n/N (%)	**NH,** n/N (%)	***p*-Value; OR [95% CI]**	***Sensitivity analysis* *p*-Value; OR [95% CI]**
**SUBACUTE/DYNAMIC EYES**								
CRB	60/142 (42.3)	40/193 (20.7)	0.002; 2.29 [1.35; 3.88]	0.008; 2.10 [1.22; 3.64]	64/121 (52.9)	27/75 (36.0)	0.030; 2.08 [1.07; 4.10]	0.048; 1.96 [1.01; 3.89]
CRR	47/142 (33.1)	35/193 (18.1)	0.087; 1.65 [0.93; 2.92]	0.215; 1.45 [0.80; 2.62]	58/121 (47.9)	25/75 (33.3)	0.068; 1.86 [0.96; 3.66]	0.113; 1.73 [0.88; 3.44]
CRS	20/31 (64.5)	9/40 (22.5)	<0.001; 7.32 [2.34; 25.91]	0.001; 7.80 [2.17; 33.84]	18/27 (66.7)	6/13 (46.2)	0.096; 5.23 [0.76; 52.91]	0.037; 12.53 [1.15; 375.2]
CRW	30/103 (29.1)	76/130 (58.5)	<0.001; 0.33 [0.18; 0.61]	0.023; 0.45 [0.22; 0.89]	24/93 (25.8)	25/49 (51.0)	0.005; 0.30 [0.12; 0.70]	0.009; 0.29 [0.11; 0.74]
**CHRONIC EYES**								
CRB	72/143 (50.3)	59/153 (38.6)	0.009; 1.93 [1.18; 3.17]	0.009; 1.94 [1.18; 3.21]	57/116 (49.1)	35/93 (37.6)	0.018; 2.05 [1.13; 3.79]	0.022; 2.05 [1.11; 3.87]
CRR	47/143 (32.9)	30/153 (19.6)	0.003; 2.24 [1.30; 3.93]	0.006; 2.16 [1.25; 3.80]	37/116 (31.9)	15/93 (16.1)	0.001; 3.15 [1.55; 6.77]	0.005; 2.84 [1.36; 6.24]
CRS	32/34 (94.1)	36/38 (94.7)	0.894; 0.85 [0.06; 10.23]	0.919; 0.86 [0.04; 15.18]	26/28 (92.9)	22/23 (95.7)	0.870; 0.75 [0.01; 20.38]	0.908; 0.55 [0.00; 3372]
CRW	4/81 (4.9)	15/89 (16.9)	0.006; 0.22 [0.06; 0.66]	0.016; 0.26 [0.07; 0.78]	2/68 (2.9)	12/60 (20.0)	<0.001; 0.08 [0.01; 0.34]	<0.001; 0.09 [0.01; 0.40]

CI: confidence interval.; CRB: clinically relevant benefit; CRR: clinically relevant recovery; CRS: clinically relevant stabilization; CRW: clinically relevant worsening; ide: idebenone; mITT: modified Intention-To-Treat; NH: Natural History cohort; OR: odds ratio.

Table S 6: Logistic regression Type III tests of Fixed Effects for CRB at 12 and 24 months in subacute/dynamic and chronic eyes by mutation (mITT vs matched NH group), related to Figure 1

Interaction model	Month 12 *p*-values	Month 24 *p*-values
**Subacute/dynamic eyes**		
Interaction treatment and major 3 mutations	0.0015	0.0078
Treatment	0.0788	0.4529
Mutations	<.0001	<.0001
Gender	0.1726	0.0021
**Chronic eyes**		
Interaction treatment and major 3 mutations	0.0242	0.0260
Treatment	0.5716	0.9798
Mutations	<.0001	0.0232
Gender	0.3358	0.3243

Table S 7. Responder analysis outcomes in subacute/dynamic and chronic eyes by mutation (mITT vs matched NH group), related to Figure 1

	**m.11778G>A**						**m.3460G>A**						**m.14484T>C**					
	**12 months**			**24 months**			**12 months**			**24 months**			**12 months**			**24 months**		
	**Ide,** n/N (%)	**NH,** n/N (%)	***p*-Value; Odds Ratio [95% CI]**	**Ide** n/N (%)	**NH,** n/N (%)	***p*-Value; Odds Ratio [95% CI]**	**Ide,** n/N (%)	**NH,** n/N (%)	***p*-Value; Odds Ratio [95% CI]**	**Ide,** n/N (%)	**NH,** n/N (%)	***p*-Value; Odds Ratio [95% CI]**	**Ide,** n/N (%)	**NH,** n/N (%)	***p*-Value; Odds Ratio [95% CI]**	**Ide,** n/N (%)	**NH,** n/N (%)	***p*-Value; Odds Ratio [95% CI]**
**SUBACUTE/DYNAMIC EYES**																		
CRB	25/68 (36.8)	13/138 (9.4)	<0.001; 6.01 [2.85; 13.24]	27/60 (45.0)	8/47 (17.0)	<0.001; 5.30 [2.12; 14.49]	12/32 (37.5)	14/34 (41.2)	1.0; 1.00 [0.36; 2.77]	9/26 (34.6)	11/18 (61.1)	0.24; 0.47 [0.12; 1.67]	23/42 (54.8)	13/21 (61.9)	0.60; 0.75 [0.25; 2.18]	28/35 (80.0)	8/10 (80.0)	0.98; 1.02 [0.13; 5.52]
CRR	14/68 (20.6)	10/138 (7.2)	0.005; 3.54 [1.48; 8.75]	23/60 (38.3)	7/47 (14.9)	0.002; 4.51 [1.75; 12.84]	12/32 (37.5)	14/34 (41.2)	0.99; 1.00 [0.36; 2.77]	9/26 (34.6)	11/18 (61.1)	0.22; 0.45 [0.12; 1.61]	21/42 (50.0)	11/21 (52.4)	0.87; 0.92 [0.32; 2.64]	26/35 (74.3)	7/10 (70.0)	0.77; 1.27 [0.23; 5.87]
CRS	12/15 (80.0)	3/28 (10.7)	<0.001; 37.03 [7.13; 283.2]	9/12 (75.0)	1/6 (16.7)	<0.001; 127E4 [6.84; N.E.]	0/6 (0.0)	2/6 (33.3)	0.10; 0.00 [N.E.; 1.81]	0/6 (0.0)	2/4 (50.0)	0.09;0.00 [N.E.; 1.70]	8/10 (80.0)	4/6 (66.7)	0.63; 1.78 [0.15; 20.80]	9/9 (100.0)	3/3 (100.0)	N.E.; 1.0 [N.E.; N.E.]
CRW	10/45 (22.2)	64/92 (69.6)	<0.001; 0.13 [0.05; 0.28]	10/41 (24.4)	20/30 (66.7)	<0.001; 0.11 [0.03; 0.34]	14/20 (70.0)	9/17 (52.9)	0.28; 2.10 [0.54; 8.59]	13/17 (76.5)	3/10 (30.0)	0.05; 5.63 [1.02; 38.94]	6/38 (15.8)	3/21 (14.3)	0.88; 1.12 [0.26; 5.83]	1/35 (2.9)	2/9 (22.2)	0.07; 0.10 [0.00; 1.19]
**CHRONIC EYES**																		
CRB	50/105 (47.6)	24/102 (23.5)	<0.001; 2.93 [1.63; 5.40]	36/82 (43.9)	11/51 (21.6)	0.008; 2.85 [1.31; 6.56]	10/23 (43.5)	16/26 (61.5)	0.24; 0.51 [0.16; 1.58]	10/23 (43.5)	16/24 (66.7)	0.12; 0.40 [0.12; 1.28]	12/15 (80.0)	19/25 (76.0)	0.81; 1.21 [0.26; 6.60]	11/11 (100.0)	8/18 (44.4)	<0.001; 462E4 [4.86; N.E.]
CRR	29/105 (27.6)	17/102 (16.7)	0.06; 1.90 [0.97; 3.78]	20/82 (24.4)	7/51 (13.7)	0.13; 2.03 [0.82; 5.54]	7/23 (30.4)	6/26 (23.1)	0.52; 1.52 [0.42; 5.64]	7/23 (30.4)	5/24 (20.8)	0.43; 1.70 [0.45; 6.78]	11/15 (73.3)	7/25 (28.0)	0.006; 6.86 [1.73; 32.34]	10/11 (90.9)	3/18 (16.7)	<0.001; 46.98 [5.90; 1065]
CRS	24/26 (92.3)	9/9 (100.0)	0.23; 0.00 [N.E.; 4.00]	18/20 (90.0)	4/4 (100.0)	0.45; 0.00 [N.E.; 13.59]	6/6 (100.0)	12/13 (92.3)	0.29; 30790 [0.11; N.E.]	6/6 (100.0)	12/12 (100.0)	N.E.; 1.29 [N.E.; N.E.]	2/2 (100.0)	15/16 (93.8)	0.7156; 14525 [0.01; N.E.]	2/2 (100.0)	6/7 (85.7)	0.54; 44571 [0.02; N.E.]
CRW	2/55 (3.6)	11/44 (25.0)	0.002; 0.11 [0.02; 0.46]	2/45 (4.4)	6/19 (31.6)	0.004; 0.10 [0.01; 0.49]	0/15 (0.0)	3/21 (14.3)	0.05; 0.00 [N.E.; 1.00]	0/15 (0.0)	3/23 (13.0)	0.05; 0.00 [N.E.; 1.03]	2/11 (18.2)	1/24 (4.2)	0.17; 5.55 [0.47; 129.9]	0/8 (0.0)	3/18 (16.7)	0.21; 0.00 [N.E.; 3.29]

CRB: clinically relevant benefit; CRR: clinically relevant recovery; CRS: clinically relevant stabilization; CRW: clinically relevant worsening; ide: idebenone; mITT: modified Intention-To-Treat; N.E.: not estimable; NH: Natural History cohort.

Table S 8. Visual acuity (logMAR) at baseline and in the 12- and 24-month cohorts (by eyes; mITT vs matched NH group), related to Figure 4

	**Total**		**m.11778G>A**		**m.3460G>A**		**m.14484T>C**	
	**Idebenone**	**NH**	**Idebenone**	**NH**	**Idebenone**	**NH**	**Idebenone**	**NH**
**Subacute/dynamic eyes, 12 months**								
N	142	193	68	138	32	34	42	21
**VA at baseline, logMAR**								
LS-Means (SE)	1.29 (0.04)	1.26 (0.05)	1.29 (0.06)	1.27 (0.05)	1.35 (0.09)	1.46 (0.09)	1.23 (0.08)	1.02 (0.11)
95% CI	1.20; 1.38	1.17; 1.36	1.17; 1.42	1.18; 1.37	1.18; 1.53	1.28; 1.64	1.07; 1.38	0.80; 1.23
**VA at 12 months**								
LS-Means (SE)	1.20 (0.04)	1.32 (0.05)	1.25 (0.06)	1.57 (0.04)	1.49 (0.08)	1.27 (0.08)	0.99 (0.08)	0.84 (0.11)
95% CI	1.12; 1.29	1.23; 1.42	1.13; 1.36	1.48; 1.66	1.32; 1.65	1.10; 1.43	0.84; 1.14	0.64; 1.05
**Difference, logMAR (*p*-value)[a]**	-0.12 (0.03)		-0.33 (<0.001)		0.22 (0.06)		0.14 (0.25)	
**Subacute/dynamic eyes, 24 months**								
N	121	75	60	47	26	18	35	10
**VA at baseline, logMAR**								
LS-Means (SE)	1.26 (0.05)	1.30 (0.07)	1.31 (0.06)	1.36 (0.07)	1.29 (0.09)	1.45 (0.12)	1.18 (0.09)	1.03 (0.15)
95% CI	1.17; 1.36	1.17; 1.43	1.19; 1.43	1.21; 1.50	1.11; 1.48	1.22; 1.68	1.01; 1.35	0.73; 1.33
**VA at 24 months, logMAR**								
LS-Means (SE)	1.11 (0.05)	1.14 (0.08)	1.22 (0.07)	1.53 (0.08)	1.48 (0.10)	0.95 (0.13)	0.77 (0.09)	0.57 (0.17)
95% CI	1.00; 1.22	0.98; 1.29	1.08; 1.35	1.37; 1.69	1.28; 1.68	0.70; 1.20	0.58; 0.95	0.23; 0.90
**Difference, logMAR (*p*-value)[a]**	-0.03 (0.75)		-0.32 (0.002)		0.53 (0.001)		0.20 (0.29)	
**Chronic eyes, 12 months**								
N	143	153	105	102	23	26	15	25
**VA at baseline, logMAR**								
LS-Means (SE)	1.16 (0.06)	1.11 (0.06)	1.35 (0.06)	1.49 (0.06)	1.31 (0.11)	0.82 (0.11)	1.25 (0.14)	0.71 (0.11)
95% CI	1.04; 1.27	1.00; 1.22	1.23; 1.46	1.38; 1.60	1.09; 1.53	0.61; 1.04	0.97; 1.53	0.49; 0.92
**VA at 12 months, logMAR**								
LS-Means (SE)	1.11 (0.03)	1.21 (0.03)	1.17 (0.03)	1.27 (0.03)	1.16 (0.06)	1.20 (0.06)	0.97 (0.08)	1.17 (0.06)
95% CI	1.05; 1.18	1.15; 1.28	1.10; 1.23	1.21; 1.34	1.04; 1.29	1.07; 1.33	0.82; 1.13	1.04; 1.30
**Difference, logMAR (*p*-value)[a]**	-0.10 (0.004)		-0.10 (0.02)		-0.04 (0.68)		-0.20 (0.05)	
**Chronic eyes, 24 months**								
N	116	93	82	51	23	24	11	18
**VA at baseline, logMAR**								
LS-Means (SE)	1.20 (0.07)	1.18 (0.07)	1.34 (0.07)	1.54 (0.08)	1.32 (0.11)	0.82 (0.11)	1.21 (0.17)	1.00 (0.13)
95% CI	1.07; 1.33	1.04; 1.31	1.21; 1.47	1.38; 1.69	1.10; 1.54	0.60; 1.04	0.88; 1.54	0.75; 1.25
**VA at 24 months, logMAR**								
LS-Means (SE)	1.07 (0.04)	1.24 (0.04)	1.15 (0.04)	1.26 (0.05)	1.11 (0.06)	1.24 (0.07)	0.75 (0.09)	1.27 (0.07)
95% CI	0.99; 1.15	1.16; 1.31	1.08; 1.23	1.17; 1.36	0.98; 1.24	1.11; 1.37	0.57; 0.94	1.13; 1.42
**Difference, logMAR (*p*-value)[a]**	-0.17 (<0.001)		-0.11 (0.04)		-0.13 (0.16)		-0.52 (<0.001)	

[a]Difference in LS-means VA in change from baseline between idebenone-treated eyes and matched eyes in the NH group. A negative difference indicates a relative improvement in visual acuity in treated eyes compared to the Natural History group.

CI: confidence interval; LS: least squares; mITT: modified Intention-To-Treat cohort; NH: Natural History cohort; SE: standard error; VA: visual acuity.

Table S 9. Safety population baseline demographics, related to Table 1

	Safety population (N=198)
Gender, N	198
Male, n (%)	146 (73.7)
Female, n (%)	52 (26.3)
Childbearing potential, N	52
Yes, n (%)	35 (67.3)
No, n (%)	17 (32.7)
Mutation, N	198
m.11778G>A, n (%)	112 (56.6)
m.3460G>A, n (%)	35 (17.7)
m.14484T>C, n (%)	34 (17.2)
Other, n (%)	5 (2.5)
Negative, n (%)	12 (6.1)
Race, N	198
Black or African American, n (%)	9 (4.5)
White, n (%)	54 (27.3)
Other[a], n (%)	135 (68.2)
Height at baseline (cm), N	191
Mean (SD)	171.3 (11.3)
Min – max	107.2 – 195.0
Weight at baseline (kg), N	191
Mean (SD)	73.6 (15.7)
Min – max	34.1 – 119.8
Age at baseline (years), N	198
Mean (SD)	34.2 (15.2)
Min – max	12.13 – 79.2

[a]Other races included 2 (1.0%) American Indian or Alaska Native, 1 (0.5%) Asian Indian, 1 (0.5%) Chinese, and 1 (0.5%) Filipino. For 130 (65.7%) patients the race was not specified.
max: maximum; min: minimum; SD: standard deviation.

Table S 10. Summary of treatment-emergent adverse events (safety population), related to Table 2

	Events	Patients	Days in Treatment	
	f (%) (F=891)	n (%) (N=198)	Mean (SD)	Min – Max
Any TEAEs	891 (100.0)	154 (77.8)	247.0 (234.9)	1.0 – 1027.0
Product-related TEAEs	101 (11.3)	49 (24.7)	120.4 (178.5)	1.0 – 757.0
Severe TEAEs	25 (2.8)	13 (6.6)	325.0 (252.5)	19.0 – 742.0
Serious TEAEs not leading to death	44 (4.9)	27 (13.6)	291.4 (239.7)	14.0 – 742.0
TEAEs (special interest)	79 (8.9)	36 (18.2)	353.5 (291.3)	1.0 – 1027.0
TEAEs leading to permanent study treatment discontinuation	18 (2.0)	10 (5.1)	241.3 (264.6)	14.0 – 742.0
TEAEs leading to death	1 (0.1)	1 (0.5)	517.0 (-)	517.0 – 517.0

max: maximum; min: minimum; SD: standard deviation; TEAE: treatment-emergent adverse event.

REFERENCES

1. Yu-Wai-Man, P.; Griffiths, P. G.; Hudson, G.; Chinnery, P. F. (2009): Inherited mitochondrial optic neuropathies. In *J Med Genet* 46 (3), pp. 145–158. DOI: 10.1136/jmg.2007.054270.
